# Supplementary material for: Proteome allocations change linearly with the specific growth rate of Saccharomyces cerevisiae under glucose limitation
Source: Nat Commun. 2022 May 20;13:2819. doi: 10.1038/s41467-022-30513-2 (PMC9122918; doi:10.1038/s41467-022-30513-2)
Supplement: Supplementary file 1 — Supplementary Information [file 41467_2022_30513_MOESM1_ESM.pdf]

Supplementary Information: Proteome allocations change linearly with the specific growth rate of *Saccharomyces cerevisiae* under glucose limitation

Jianye Xia[1,2,3], Benjamin J. Sánchez[2], Yu Chen[1,2], Kate Campbell[2], Sergo Kasvandik[4], Jens Nielsen[2,5,*]

[1] State Key Laboratory of Bioreactor Engineering, East China University of Science and Technology, Shanghai, 200237, China

[2] Department of Biology and Biological Engineering, Chalmers University of Technology, Gothenburg, SE41296, Sweden

[3] Key Laboratory of Systems Microbial Biotechnology, Tianjin Institute of Industrial Biotechnology, Chinese Academy of Sciences, Tianjin, 300308, China

[4] Institute of Technology, University of Tartu, Tartu, 50411, Estonia

[5.] BioInnovation Institute, Ole Maaløes Vej 3, DK2200 Copenhagen N, Denmark

*Correspondence: nielsenj@chalmers.se

Supplementary Note 1. Statistics information about the proteome data obtained in our experiment

We did biological triplicates at each specific growth rate, which were implemented through glucose-limited chemostats. And we got the absolute proteome data for each samples (Supplementary Data 1). We then did a basic statistical analysis with our proteome datasets. It was found that 2,821 proteins were detected at once for all 27 samples (9 specific growth rates with 3 sample for each), and 1,787 proteins were detected among all the 9 studied specific growth rates (with at least two times detected in all triplicate samples). There were 137 proteins that were only detected under a specific growth rate, and these proteins were listed as bellow in the Supplementary Table 1.

Supplementary Table 1. The unique proteins that are only detected in each of the studied specific growth rate.

μ / h^{-1}	Number	Unique proteins detected
0.027	33	RAD50, THI20, SRB4, VPS35, CYR1, MAD1, PIK1, POP1, MDS3, VAM6, SRP102, SEA4, EXO84, YNL035C, PCL6, CIN2, YMR114C, HRD3, LOT6, YDR262W, VPS33, GTR2, ATG21, RRP45, ARO10, PTH2, MNR2, YFR039C, FMN1, YDR186C, PPZ2, RMR1, YSY6
0.044	17	NAB6, YHR003C, YAP1801, AAT1, MNN4, GRX3, UTR1, PIB1, MED1, YOR111W, RTS3, SSU72, GPI17, HPA2, RAM1, TDA2, YIP5
0.102	12	INO80, RIM2, SWC4, ATG3, SNL1, PDR17, JSN1, VPH2, YKL069W, VPS41, YLR241W, RTG1
0.152	10	HEH2, POL12, YPR091C, LOA1, SPT8, YMR147W, VPS51, LSM5, ARL3, LCP5
0.214	10	MLP2, SEC15, NST1, TAO3, HDA1, NCB2, CDC36, GFD1, PCC1, SOK2
0.254	13	YOL087C, DUG2, CCM1, TSR4, YCK2, HDA2, PBP2, PSY2, ARL1, CDC8, UBC5, SEE1, YUH1
0.284	18	POL2, DAL2, TFC3, GEA1, TFB1, CCA1, RPC37, YJR149W, GAL83, MRM1, MCM5, UTR4, SEC6, MAK3, MCT1, SAM35, OCA4, FCF1
0.334	9	RPC82, YJL213W, GUF1, SIF2, YPP1, VTC3, STB1, SMD1, CEF1
0.379	15	ISM1, IMA1, PDR15, SIS2, DUS1, RCM1, NUP100, SAL1, SAP190, HSM3, RPC31, NAT3, RRP36, DBF20, IES4

Six proteins were detected only under purely respiratory metabolism condition, which are IDP3, FMP40, MDR1, YIL055C, YDR476C, AIM3; while three proteins were detected only under respire-fermentative condition, which are SCS7, TRZ1 and NOC4.

Supplementary Table 2. Linear correlation between 18 selected mRNAs absolute concentrations and their corresponding FPKM values.

Genes	Log10 absolute concentration	Log10 FPKM
YKL166C	0.941901	1.408673
YMR037C	1.283648	1.760375
YGL248W	1.084783	1.8142
YJL196C	0.997079	1.951155
YPL203W	1.146113	2.123908
YCR005C	1.292791	2.086909
YMR169C	0.519345	2.108885
YNR016C	1.914004	2.360884
YGL205W	1.553753	2.508488
YKL148C	1.889795	2.623546
YHR183W	2.009526	2.743294
YFR053C	2.257163	3.029681
YGL055W	1.898622	3.322376
YLR044C	2.461568	3.423363
YHR007C	2.108334	3.570917
YKL060C	2.379701	3.884723
YBR072W	3.016487	4.271733
YGR192C	3.067624	4.174969

Supplementary Note 2. Estimation of the translation speed of ribosome in *S. cerevisiae*

For better interpreting the observed model between ribosome protein fraction and specific growth rate (Eqn. 1 in main text), the equation is transformed to the following format,

$$\mu = \tau(f_r - f_r^0) \quad (1)$$

With $\tau = 2.86\ h^{-1}$ and $f_r^0 = 0.13$, here τ is interpreted as ribosome specific translation rate, and $\tau = 2.86\ h^{-1}$ means 2.86 gram of new protein will be translated per gram ribosome per hour, and f_r^0 means the minimum ribosome fraction in the proteome required for maintaining the cell alive when it has no growth. Then the average amino acid molecular weight was calculated to be 128.65 Da according to the amino acid compositions of all proteins detected under all 9 specific growth rate experiments (Supplementary Data 4). Given the ribosomal protein molecular weight to be 1.62×10^6 Da[1], the peptide elongation rate (PER) was estimated to be,

$$\tau = \frac{2.86\ \text{gram protein}}{gram\ \ ribosome \cdot hour} = \frac{2.86/128.65}{\frac{1}{1.62\times10^6}\cdot 3600\ sec} \approx 10\frac{\text{AA}}{\text{Ribosome}\cdot\text{sec}}$$

this agrees with 2.8-10 AA/Ribosome/s reported in Bionumbers database[2] and 10.5 AA/Ribosome/s in Waldron et al.[3].

Supplementary Note 3. Statistics information about the transcriptome data obtained in our experiment

Using the absolute mRNA quantification method, we quantified 5401 mRNAs in the 9 tested specific growth rate conditions with unit of copies per cell and shown in Supplementary Data 3. The most abundant mRNA among all 9 conditions is the translational elongation factor EF-1 alpha, TEF1, with an average value of 436 copies per cell. And the less abundant mRNA is the function unknown protein, YL138C, with an average value of 0.16 copies per cell.

Supplementary Note 4. GO terms enrichment analysis for significantly correlated 845 genes.

BP (biological process), MF (molecular function), and CC (cellular component) enrichment analysis were carried out for 845 genes which showed significant correlations in between mRNA and protein level. The results is shown in Supplementary Figure 4. From the results, it is shown that for BP enrichment analysis, the top three terms are structural constituent of ribosome with corrected p value = 1.97×10^{-37}, structural molecule activity with corrected p value = 3.99×10^{-17} and rRNA bindin g with corrected p value = 3.2×10^{-4}, respectively; for BP enrichment analysis, the top three terms are translation with corrected p value = 1.34 × 10-37.

Supplementary Note 5. Details of the Bayes inference method and inference results analysis

Strategy for inferencing the data-dependent-best fit model. According to the definition of intrinsic turn over number of a_i, the bigger of a_i the more the corresponding metabolite will influence the enzyme's k_{cat}. Here, we assume that enzymes take as less metabolite as possible to influence their k_{cat} value. With this assumption, we proposed the following strategy to get the plausible best fit model depending on the data in hand, what is called data-dependent-best fit model.

ALGORITHM 1: Inference of the data-dependent-best fit kinetic model for a bio-reaction

Input: FBA results of flux vector with component at each experimental condition (J_{obs}^{c}), absolute enzyme concentration vector at each condition (e^{c}), FVA results upper and lower flux vector with components under each condition ($J_{obs}^{c,upper}$,$J_{obs}^{c,lower}$), relative abundance of each metabolites take part in the reaction as respect to a reference condition ($\frac{X_i^c}{X_i^0}$), candidate metabolite list that may have effect on the k_{cat} (or in another word, the intrinsic turn over number of this metabolite cannot be omitted)

Output: The "best" fit model in which the most possible list of a_i were given, and their posterior distribution.

Initialization: Set all metabolites that take part in the reaction as candidate metabolite list ($\mathbf{Met}_{candidate}$); set current best fit model chosen metabolites ($\mathbf{Met}_{curr,best}$) list to be empty; set current best fit model $\mathbf{Mod}_{b,curr}$ to be empty.

LOOPing: for each metabolite **m** in $\mathbf{Met}_{candidate}$ do

Set a_b as already confirmed model paramters, for first round loop it is empty

Set model structure ($\mathbf{Mod}_m$) as $j^c = j^0 + a_m \ln(X_m^c / X_m^0) + \sum_{b=1}^{N_b} a_b \ln(X_b^c / X_b^0)$

Do MCMC Bayes inference for model $\mathbf{Mod}_m$

Do next step

Test using Leave One Out (LOO) cross-validation criteria to select best from among all $\mathbf{Mod}_m$, comparing the select out best model $\mathbf{Mod}_{m,best}$ to $\mathbf{Mod}_{b,curr}$.

If Modm,best is not better than Modb,curr:

Return $\mathbf{Mod}_{b,curr}$

else:

Append the best model corresponding metabolite m to $\mathbf{Met}_{curr,best}$

Remove m from $\mathbf{Met}_{candidate}$

Goto **LOOPing** do next round loop.

Bayes inference results analysis. Using the above MCMC Bayes inference algorithm, we do best-fitted model inference for each reaction step in the central carbon metabolism first (Supplementary Table 3). There are 28 reaction steps in the central carbon metabolism pathway (10 from EMP, 10 from TCA and 8 from HMP), and it was found that among all the 9 studied dilution rates, FBA results showed flux through the reaction step catalyzed by transaldolase in HMP were all zero, even though the corresponding enzyme protein were detected under all conditions. When doing Bayes inference modelling, this reaction step was not considered as no reaction flux information for it. The above Bayes inferences strategy (ALGORITHM 1) were carried out on the other 27 reactions, and 20 of them got fitted using the proposed model (Eqn. 8 in main text), and the best fitted model structures were inferenced based on the observed fluxome, proteome and metabolome data, respectively.

Among all the test 27 reactions, not all metabolites of each reaction were detected in our metabolome data. As it cannot distinguish water generated in reaction and that in the solution, relative change of water amount was not considered in our model. As all experiment were carried out under steady state, CO_2 level in system was considered to be constant and quick equilibrium between extra- and intra-cellular level of CO_2 was believed to be the case, thus

CO_2 was also not considered in our model. We also assume stable intracellular pH level across all conditions, so there is no change of intracellular H^+ level. According to Suarez-Mendez[4], a linear relationship between the intracellular ATP level and specific growth rate is assumed. Reaction GPI (glucose-6-phosphate isomerase) and TPI (triose-phosphate isomerase) are considered to be under equilibrium, and K_{eq} values of 0.259 (GPI) and 0.039 (TPI) taken from data in Canelas, et al.[5] are applied to get abundance information of F6P and GAP. Other missing data of metabolites are taken as no-change across all conditions. Missing metabolites for each tested reaction step were listed in Supplementary Table 3.

Supplementary Table 3. Reactions (central carbon metabolism) considered in the Bayes inference modelling. Missing data on metabolites and reported regulators are listed for each reaction.

Pathway	Reaction	Missing data on metabolites[1]	Regulators reported[6]
EMP	Hexokinase	ATP	
	glucose-6-phosphate isomerase	F6P	
	phosphofructokinase	ATP	3PG(-),isoCIT(-), PEP(-),FBP(+)
	fructose-bisphosphate aldolase	GAP,	ADP(-), AMP(-), CIT(-), E4P(-), dATP(+), HISnol(+)
	triose-phosphate isomerase	GAP	AMP(-), GTT(-), GTTdS(-), ASP(+)
	glyceraldehyde-3-phosphate dehydrogenase	GAP	
	phosphoglycerate kinase	13PG, ATP	
	phosphoglycerate mutase	-	
	enolase	-	
	pyruvate kinase	ATP	MAL(-), aKG(-), 3PG(-), AMP(-), CIT(-), isoCIT(-), PEP(-), FBP(+), 6PG(+), DHAP(+), GAP(+), ALA(+), ASP(+), GLU(+), GLY(+), ILE(+), MET(+), R5P(+)
HMP	glucose 6-phosphate dehydrogenase	NADP, 6PGL, NADPH	AMP(-), PEP(-)
	6-phosphogluconolactonase	6PGL	
	phosphogluconate dehydrogenase	NADP, Ru5P, NADPH	ASP(+), HIS(+), TRP(+)

[1] H_2O and CO2 data was not considered.

	ribulose 5-phosphate 3-epimerase	Ru5P, Xu5P,	
	ribose-5-phosphate isomerase	Ru5P	
	transketolase 1	Xu5P	
	transaldolase[2]	E4P	
	transketolase 2	E4P, Xu5P	
TCA	pyruvate carboxylase	ATP, ADP, H^+, OA	
	citrate synthase	H^+	
	citrate to cis-aconitate(3-)	Cis-CAN	
	cis-aconitate(3-) to isocitrate	Cis_CAN	
	isocitrate dehydrogenase	-	
	oxoglutarate dehydrogenase	H^+	
	succinate-CoA ligase	SUC-CoA	
	succinate dehydrogenase	SUC-CoA, ubQ	
	fumarase	-	
	malate dehydrogenase	H+	CIT(-)

According to the best fitted model searching algorithm (ALGORITHM1), there will be one best fitted model that include minimum amount of plausible allosteric effectors (here we only consider the metabolites that take part in the reaction, no other allosteric regulators were considered), e.g. For the first reaction step of EMP (the HK catalyzed reaction), the best fitted model shows that it is plausible ADP and ATP have allosteric effect on the enzyme (the HK enzyme), while allosteric effect of glucose and G6P can be omitted. The inferenced intrinsic turn over numbers of best fitted model for each of the reactions are plotted in Figure S0. It can be seen that not all participated metabolite show allosteric regulation effect for each reactions, however, ADP、ATP and phosphate show regulating the k_{cat} value for all reactions that they take part in, 7 among all the tested 27 reactions. The same conclusion is also observed for NADH for all 4 reactions it takes part in. Positive a_i value means positive regulation of the corresponding metabolites to the enzyme k_{cat} value, and vice versa. It is more confident to conclude that ADP shows positive effect on reaction HK and reaction PYC, NADH on reaction GAPD, ATP on reaction PGK, CIT on reaction CITS, while negative effect of PEP on reaction ENO, and not so strong confident on negative effect of DHAP on reaction FBA and TPI.

High accurate Bayes inference results depends on both the proposed model structure (whether we have chosen the right model for describing the observed data) and the amount of information about the system we are studying (whether enough data for accurately inferencing the proposed model). Here, the wide 95% credible interval of the results in Supplementary Figure 1 may be caused by two reasons: First, for some reactions we did not get all

[2] FBA results of flux for this reaction are zero under all conditions.

participated metabolite's abundance information, which will make the inferenced a_i value contains the contribution of non-measured metabolites (that makes the uncertainty higher for the inferenced a_i value). Second, here we only considered 9 chmostat conditions, which only leave us 8 available data for inferencing (one condition is chosen as reference), lack of observed data also limit our ability to get accurate inference for the intrinsic turn over numbers for each reaction. However, using the LOO criteria, the most possible kinetics model structure is figured out by the limited data, so we call the results as data-dependent-best fitted model inference. And the regulation effect of ATP, ADP and NADH has been highly recommended by our model inference results, which makes sense.

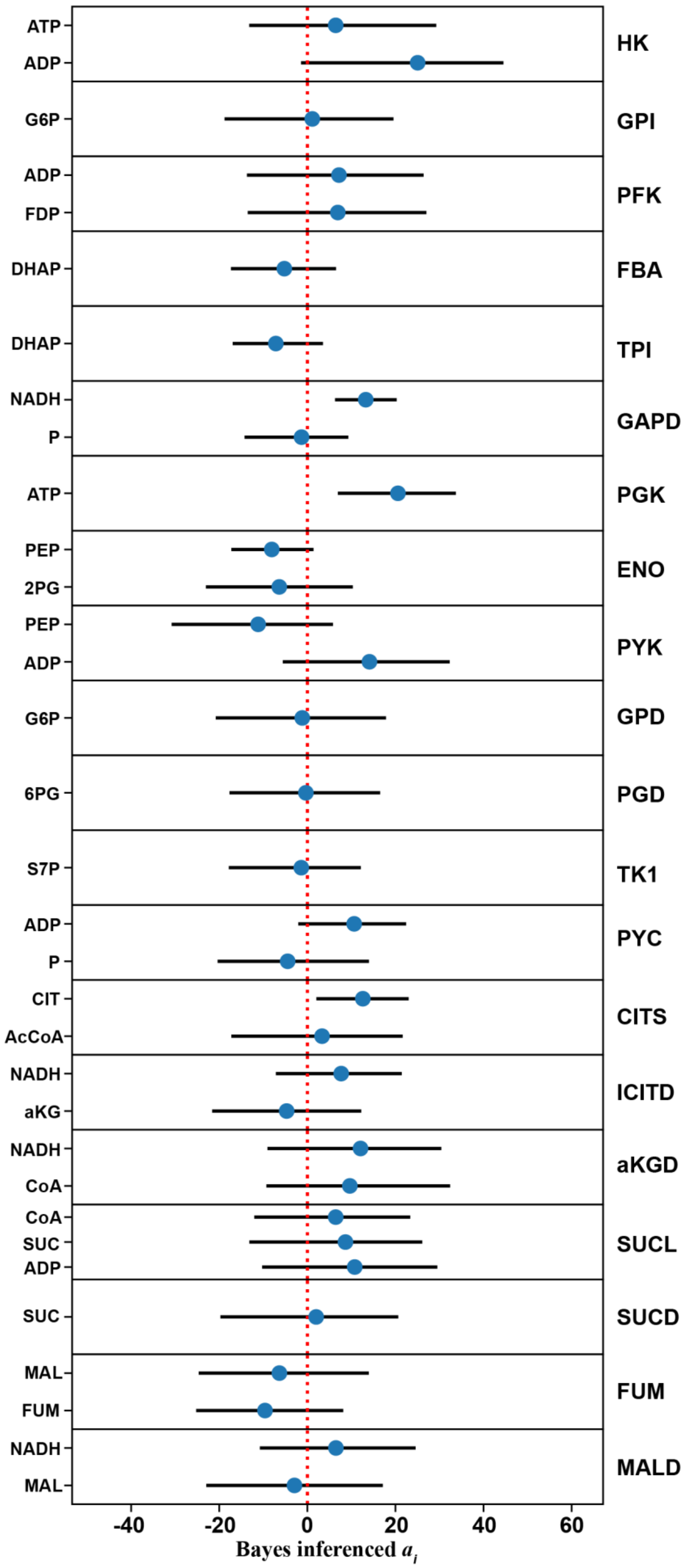
ATP
ADP
HK
G6P
GPI
ADP
FDP
PFK
DHAP
FBA
DHAP
TPI
NADH
P
GAPD
ATP
PGK
PEP
2PG
ENO
PEP
ADP
PYK
G6P
GPD
6PG
PGD
S7P
TK1
ADP
P
PYC
CIT
AcCoA
CITS
NADH
aKG
ICITD
NADH
CoA
aKGD
CoA
SUC
ADP
SUCL
SUC
SUCD
MAL
FUM
FUM
NADH
MAL
MALD
-40
-20
0
20
40
60
Bayes inferenced a_i

Supplementary Figure 1. Bayes inference results of best fitted model for central carbon metabolism pathways. Filled circle denotes mean of the posterior distribution of each intrinsic turn over number a_i from the best fitted kinetics model, and bars across each circle represents the 95% credible-interval gotten from the posterior distribution of a_i. The red vertical dotted line located at 0 is shown as a reference, a_i value higher than 0 means positive effect on the k_{cat} of enzyme, and vice versa. Abbreviations: HK: hexokinase, GPI: glucose-6-phosphate isomerase, PFK: phosphofructokinase, FBA: fructose-bisphosphate aldolase, TPI: triose-phosphate isomerase, GAPD: glyceraldehyde-3-phosphate dehydrogenase, PGK: phosphoglycerate kinase, ENO: enolase, PYK: pyruvate kinase, GPD: glucose 6-phosphate dehydrogenase, PGD: phosphogluconate dehydrogenase, TK1: transketolase 1, PYC: pyruvate carboxylase, CITS: citrate synthase, ICITD: isocitrate dehydrogenase (NAD+), aKGD: oxoglutarate dehydrogenase (dihydrolipoamide S-succinyltransferase), SUCL:succinate-CoA ligase (ADP-forming), SUCD: succinate dehydrogenase (ubiquinone-6), FUM: fumarase, MALD: malate dehydrogenase.

Supplementary Note 6. Transcription Factors (TF) analysis for glycolysis flux regulation.

Transcriptional regulation in yeast has been extensively studied earlier and several different transcription factors TFs have been identified, e.g. Gcr1 and Gcr2, but also the stress response transcription factors Msn2 and Msn4 regulate glycolytic genes[7]. However, these TFs were not observed in our proteome data. Additionally, we searched the literature and found a paper by Sierkstra et al.[8] describing the transcription level of glycolytic genes in glucose-limited chemostats with dilution rate ranging from 0.05 to 0.315 h^{-1}. But they found that most of the mRNA levels of glycolytic enzymes remained constant, while they observed that enzyme activity decrease, e.g. of phosphoglucomutase, so they concluded that there is no transcriptional or translational regulation of glycolytic flux. However, we still want to see whether some transcription factors take the responsibility to regulate the expression level of glycolytic enzymes. We checked the reported transcription factors by Lee et al.[9], in *S. cerevisiae*, and we selected 28 TF regulators and their target-genes confirmed by gene specific PCR (Table S1 in Lee et al.[9]). However, we did not find any transcription factor having their target gene to be among the glycolytic genes.

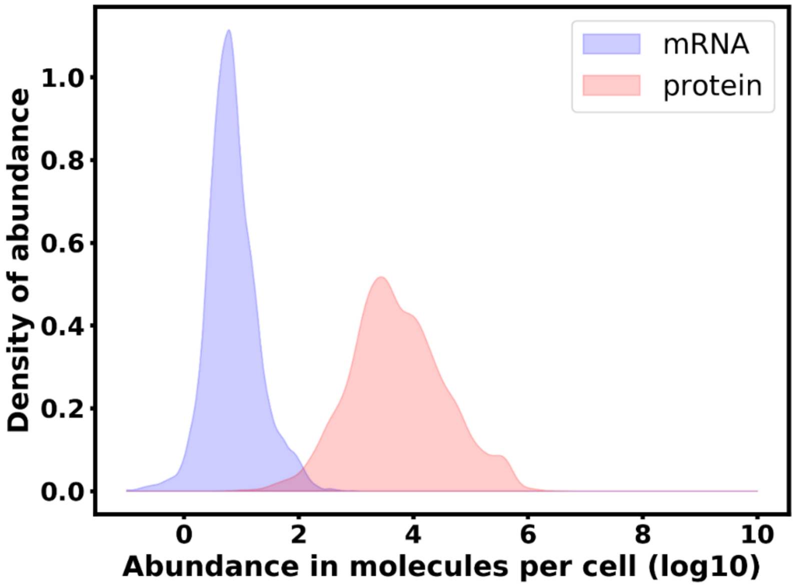

(A)

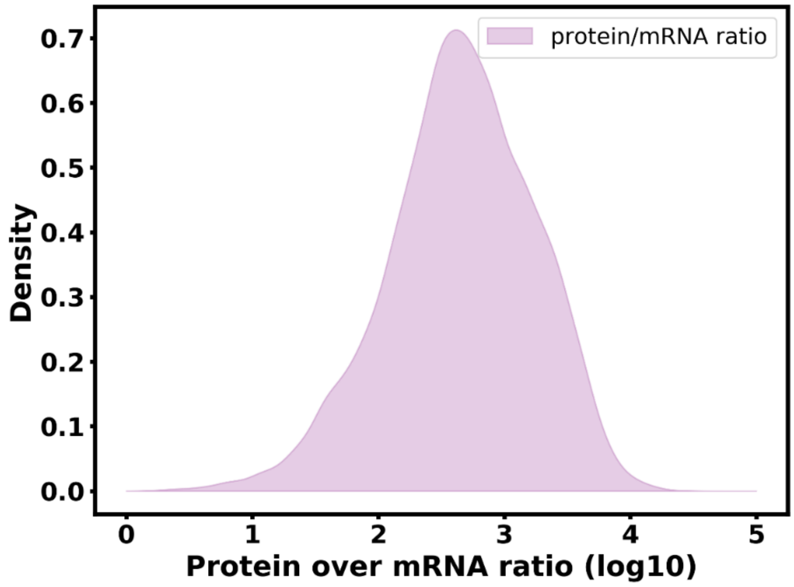

(B)

Supplementary Figure 2. Distribution of mRNA and protein abundance and their ratios across all test conditions. (A) Abundance distribution of transcriptome and proteome, in average the abundance of proteins are 3 orders bigger than that of the mRNAs. It is also noticed that few transcripts show value less than 1 copy per cell, which is caused by unequal expression among different single cells even under the steady state culture. (B) Density plot of protein over mRNA ratios with all genes detected in the 9 studied conditions.

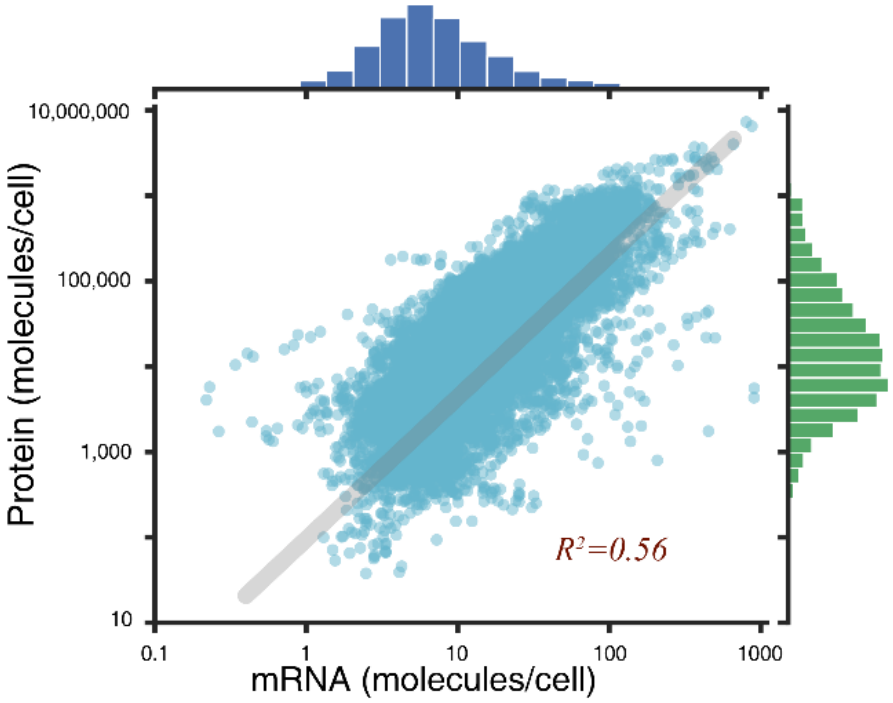

Supplementary Figure 3. Correlation between protein and mRNA abundance for all data, using all values of individual samples across different dilution rates (n=9) with biological replicates (n=3). The upper panel shows the distribution of mRNA abundance, with a median value of ~6 molecules per cell. Right panel shows the distribution of protein abundance, with a median value of ~13,500 molecules per cell. The average dry cell weight was assumed to be 13 pg according to a previous study of this strain under similar growth conditions [10].

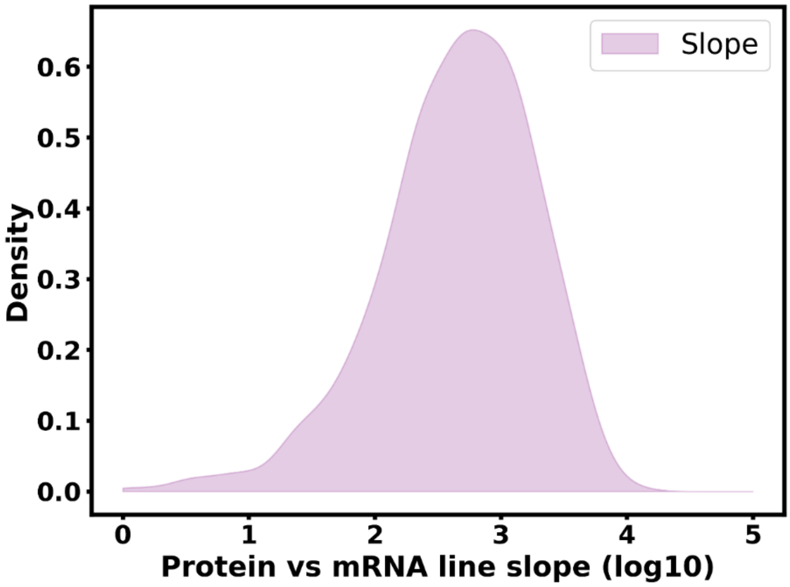

Supplementary Figure 4. Density plot of protein versus mRNA regressed line slope. The plot shows that the slopes cover three orders of magnitude among different transcripts.

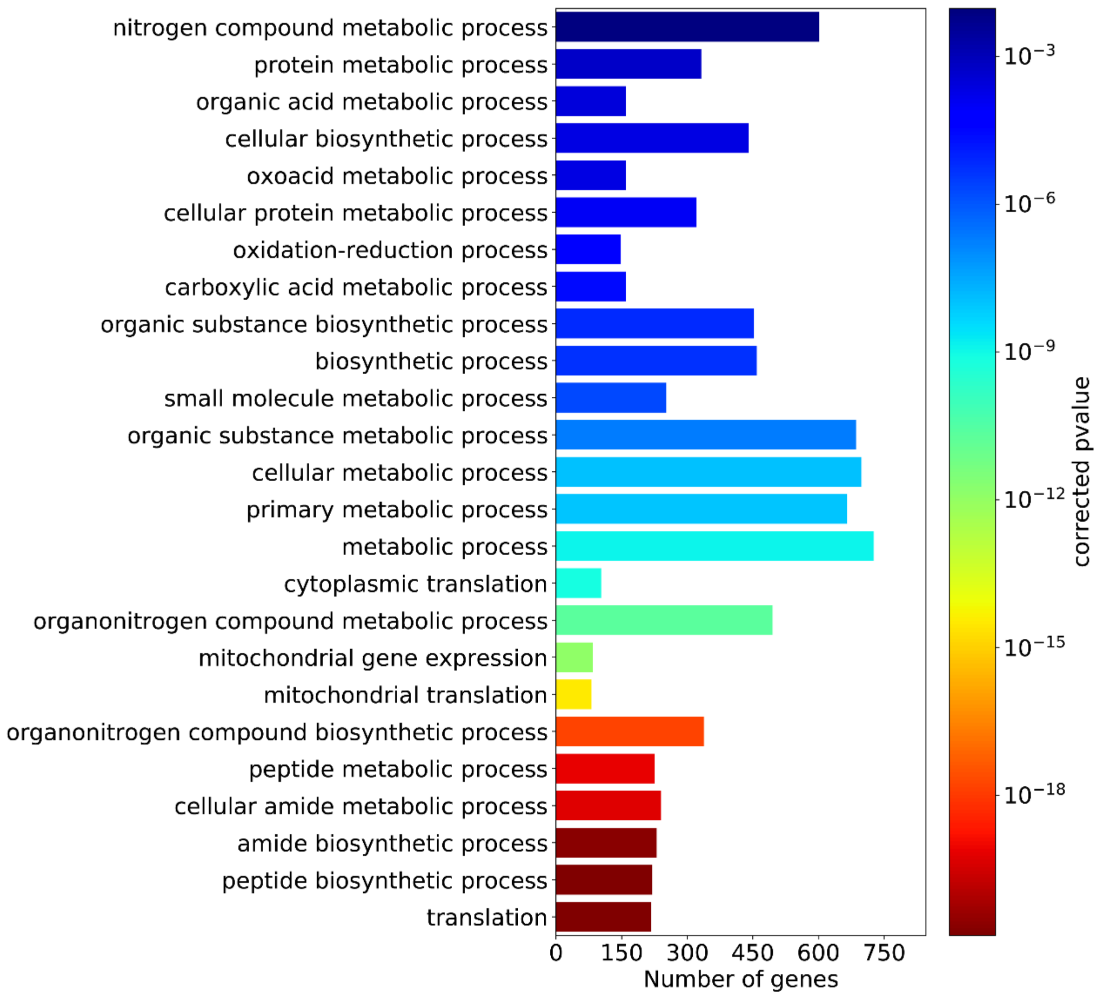
nitrogen compound metabolic process
protein metabolic process
organic acid metabolic process
cellular biosynthetic process
oxoacid metabolic process
cellular protein metabolic process
oxidation-reduction process
carboxylic acid metabolic process
organic substance biosynthetic process
biosynthetic process
small molecule metabolic process
organic substance metabolic process
cellular metabolic process
primary metabolic process
metabolic process
cytoplasmic translation
organonitrogen compound metabolic process
mitochondrial gene expression
mitochondrial translation
organonitrogen compound biosynthetic process
peptide metabolic process
cellular amide metabolic process
amide biosynthetic process
peptide biosynthetic process
translation
0 150 300 450 600 750
Number of genes
10^{-3}
10^{-6}
10^{-9}
10^{-12}
10^{-15}
10^{-18}
corrected pvalue

(A)

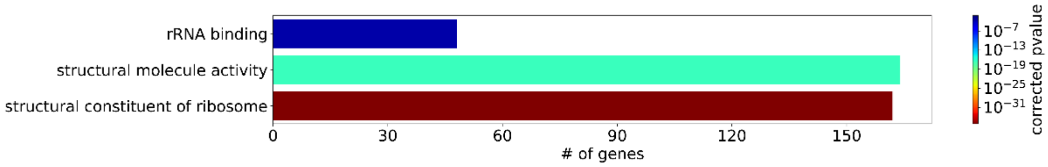

(B)

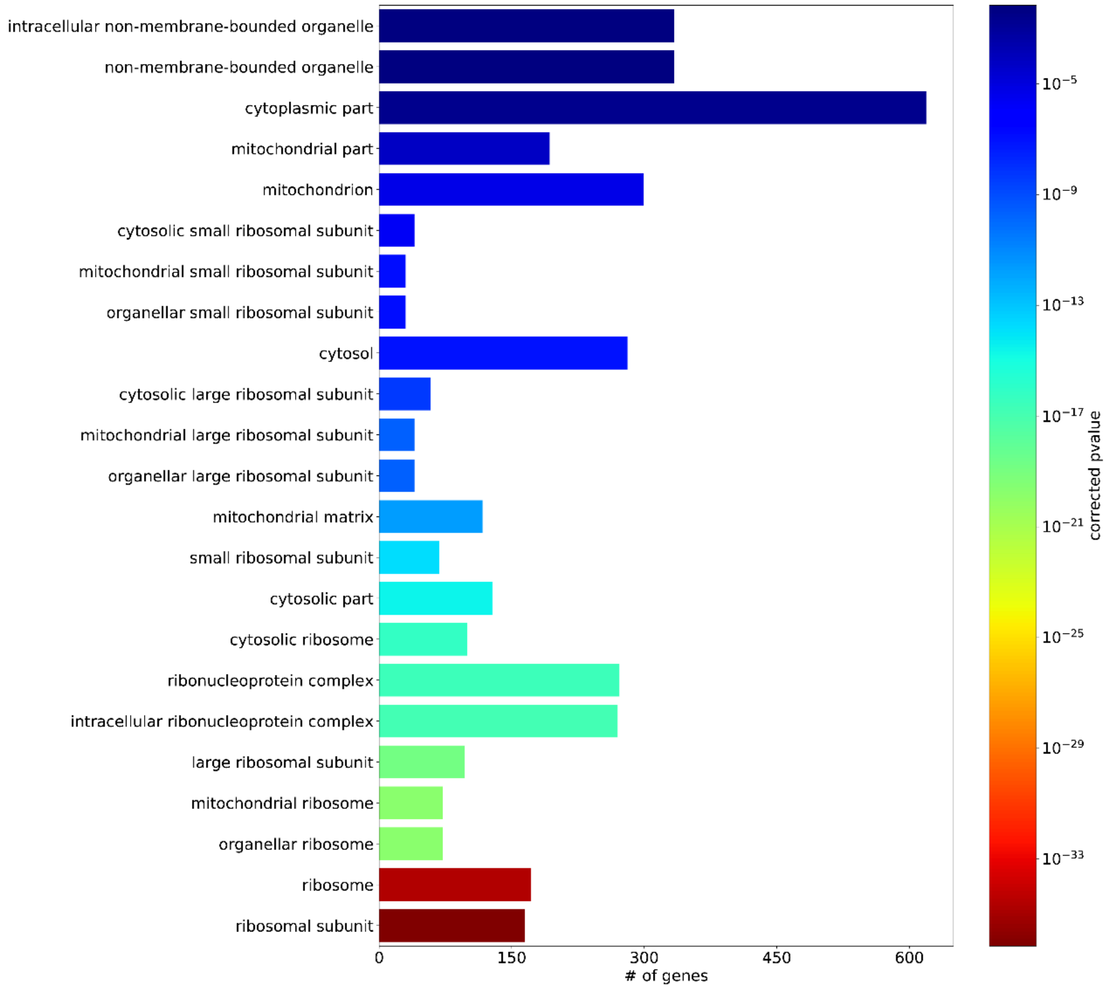

(C)

Supplementary Figure 5. GO terms Enrichment analysis of 845 genes with which significant correlations (p value < 0.01) were observed. (A) GO term biological process (BP) enrichment analysis, (B) GO term molecular function (MF) enrichment analysis, and (C) GO term cellular component (CC) enrichment analysis. Source data are provided as a Source Data file.

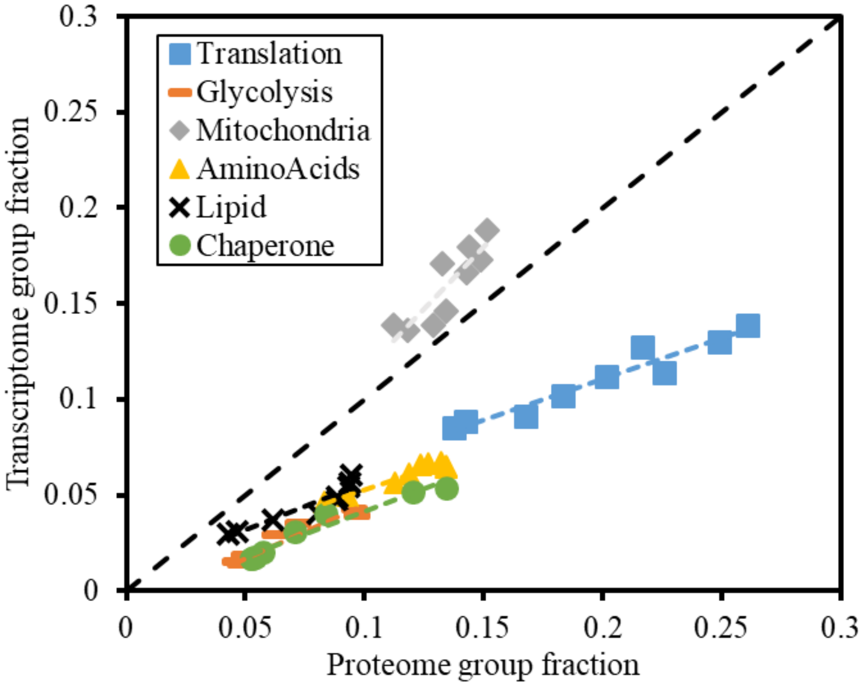

Supplementary Figure 6. **Transcriptome group fraction vs Proteome group fraction scatter plot.** The six functional groups, including translation, glycolysis, mitochondria, amino acid biosynthesis, lipid metabolism, and chaperone, were plotted, and regressed lines for each group were also shown together. The Pearson correlation coefficients for all test groups were shown in Supplementary Table 4. Source data are provided as a Source Data file.

Supplementary Table 4. Correlation analysis between transcriptome and proteome fractions for each functional group, multiple comparisons were corrected using FDR < 0.05.

Function	Correlation coefficient	pvalue	pcorr	FDR<0.05
Translation	0.967	2.14E-05	4.49E-05	TRUE
Glycolysis	0.953	7.00E-05	0.00011	TRUE
Mitochondria	0.870	0.002291	0.002406	TRUE
Amino acids	0.968	1.80E-05	4.49E-05	TRUE
Lipid	0.936	0.000206	0.00026	TRUE
Chaperone	0.975	7.63E-06	4.49E-05	TRUE

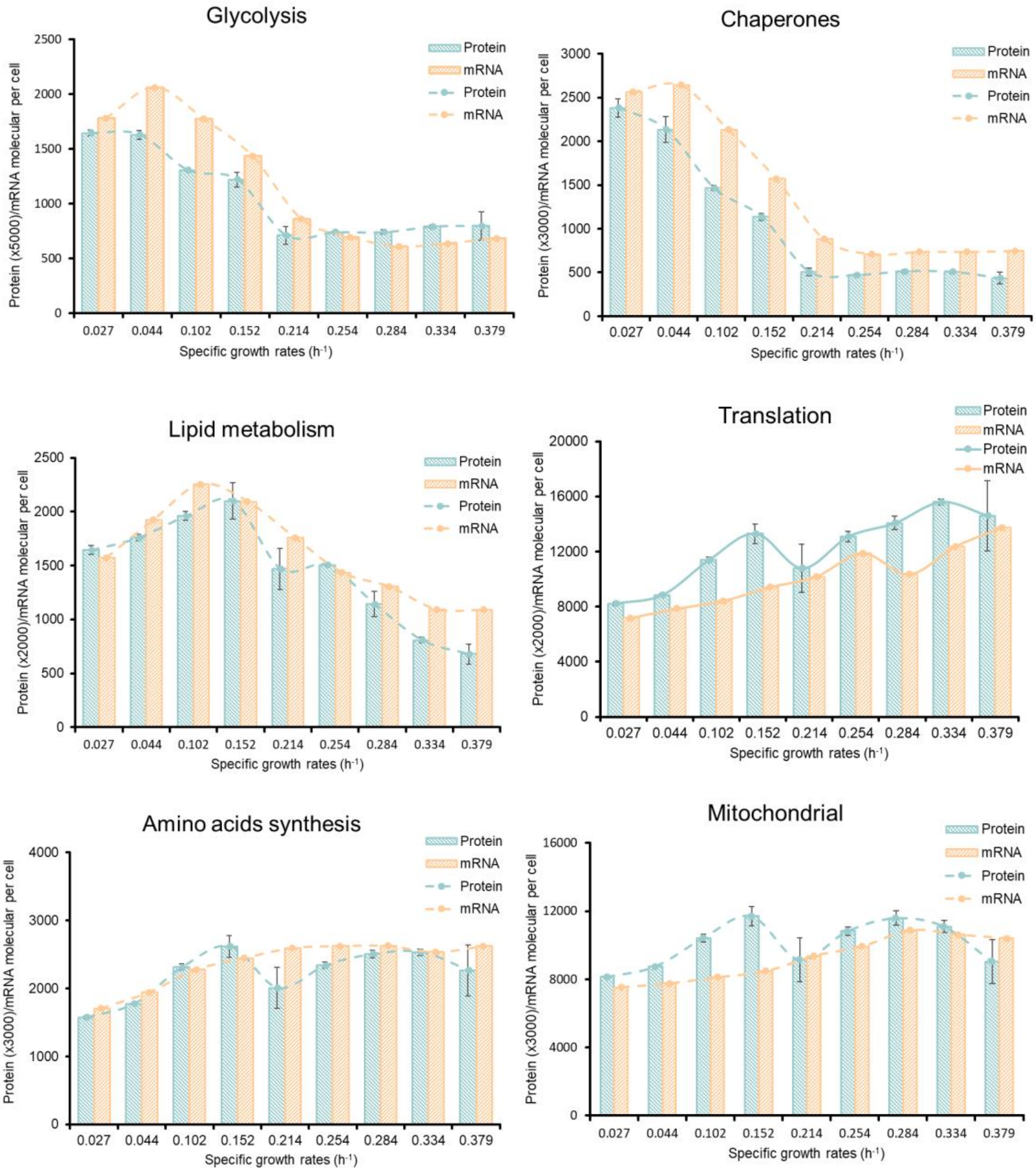

Supplementary Figure 7. The absolute concentrations of mRNA and protein under different specific growth rates (data are presented as mean ± SD with n=3 biologically independent samples). It is clear that similar trends among the absolute concentration of mRNA, protein and protein allocation in glycolysis, chaperon, translation, amino synthesis and mitochondrial functional groups. For the lipid metabolism related genes, although the mRNA and protein change trend are almost the same, it is not consistent with the protein allocation trend. Source data are provided as a Source Data file.

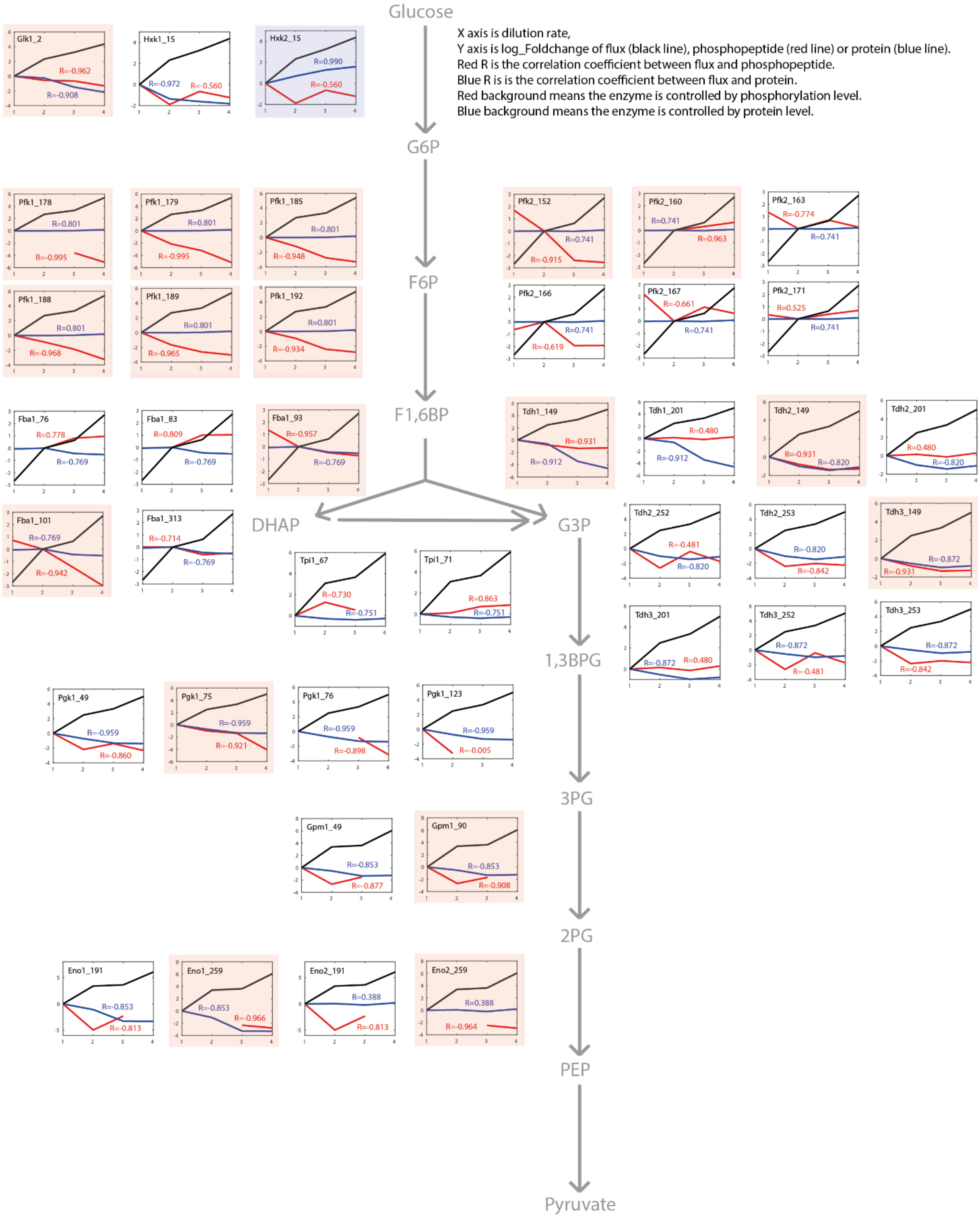

Supplementary Figure 8. Functional phosphorylation events analysis for glycolysis enzymes. Among the ten steps, seven steps' enzymes showed functional phosphorylation event sites. Source data are provided as a Source Data file.

mRNA levels at different specific rates

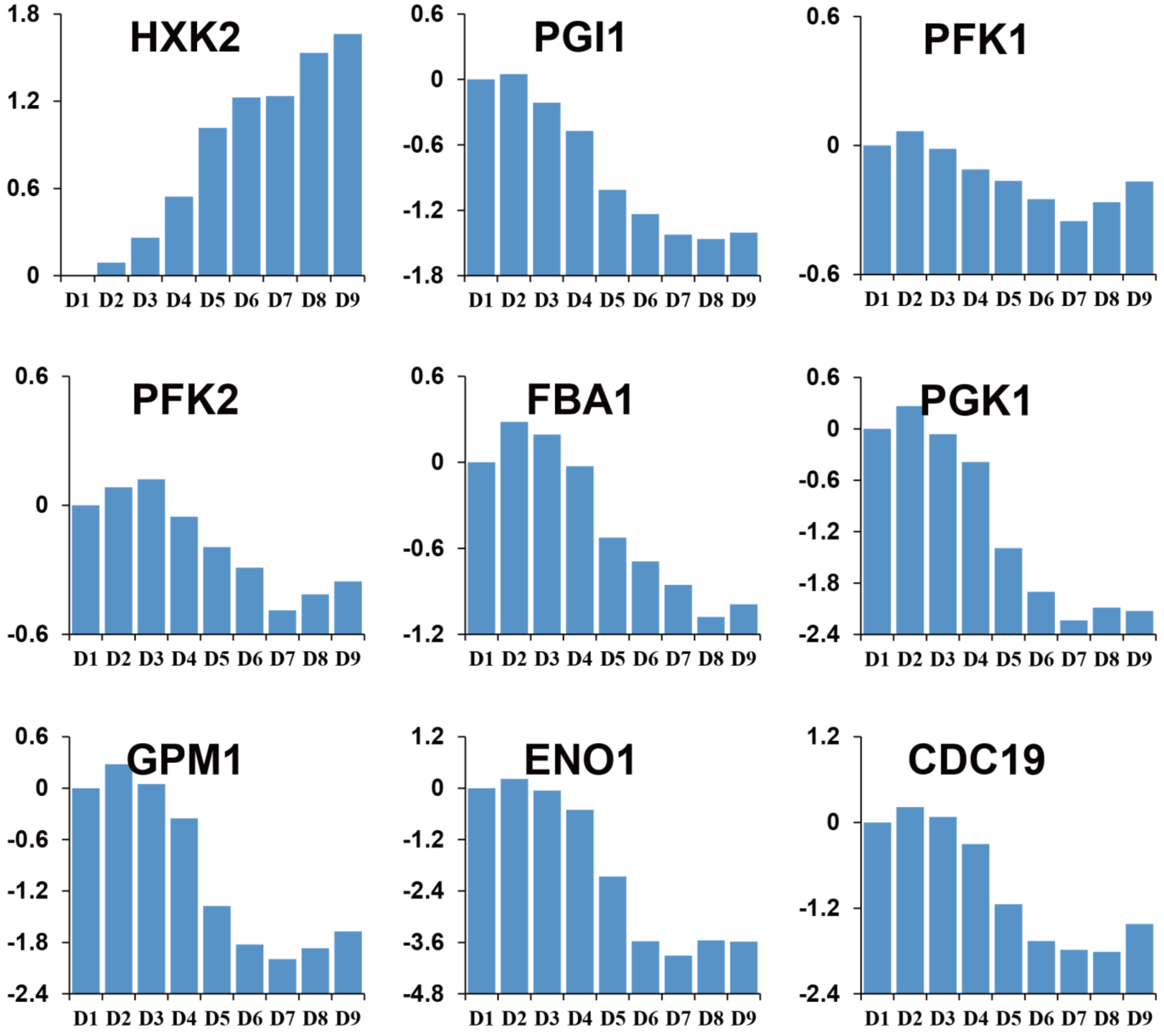

(A)

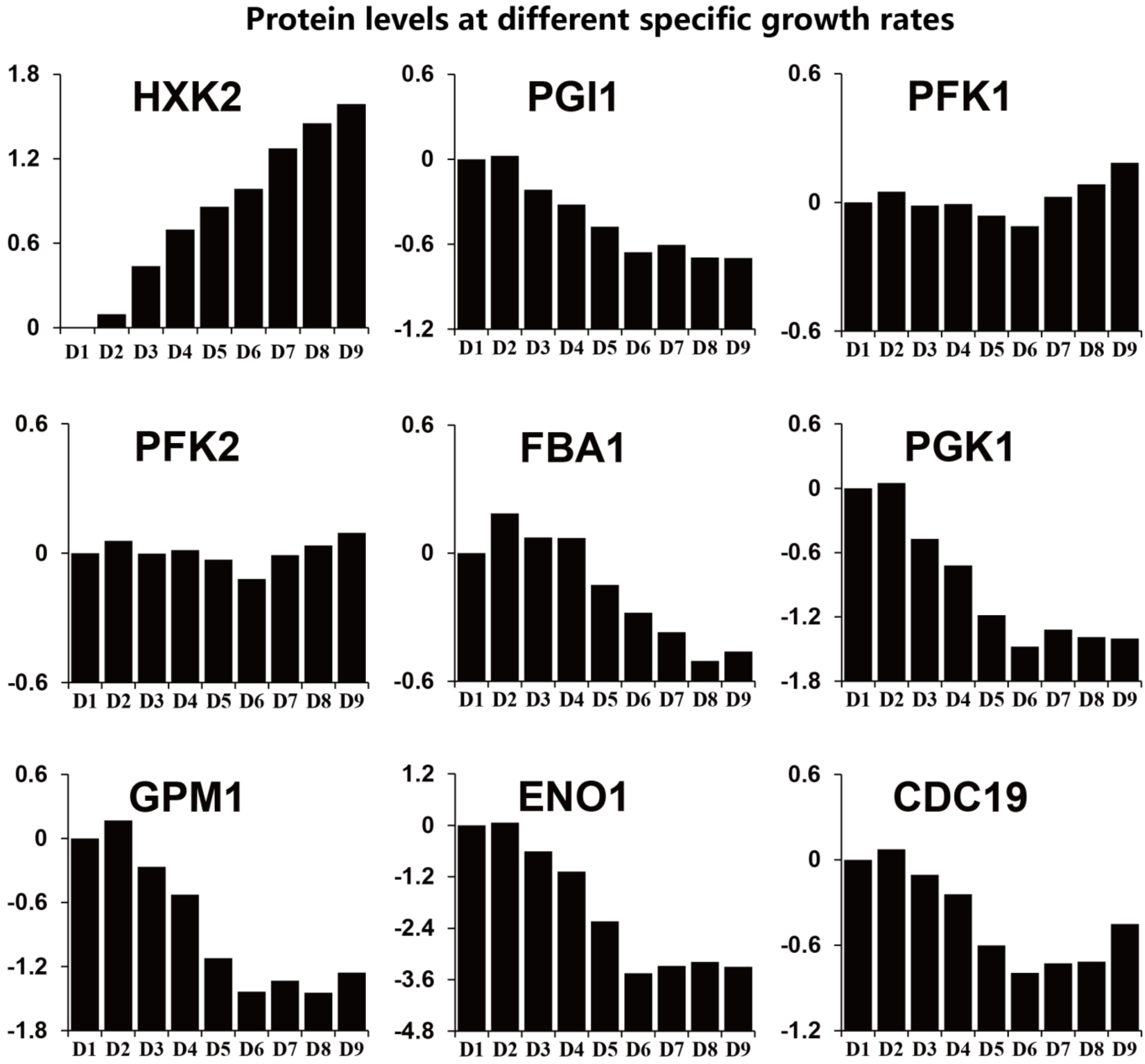
Protein levels at different specific growth rates
HXK2
PGI1
PFK1
PFK2
FBA1
PGK1
GPM1
ENO1
CDC19
D1 D2 D3 D4 D5 D6 D7 D8 D9

(B)

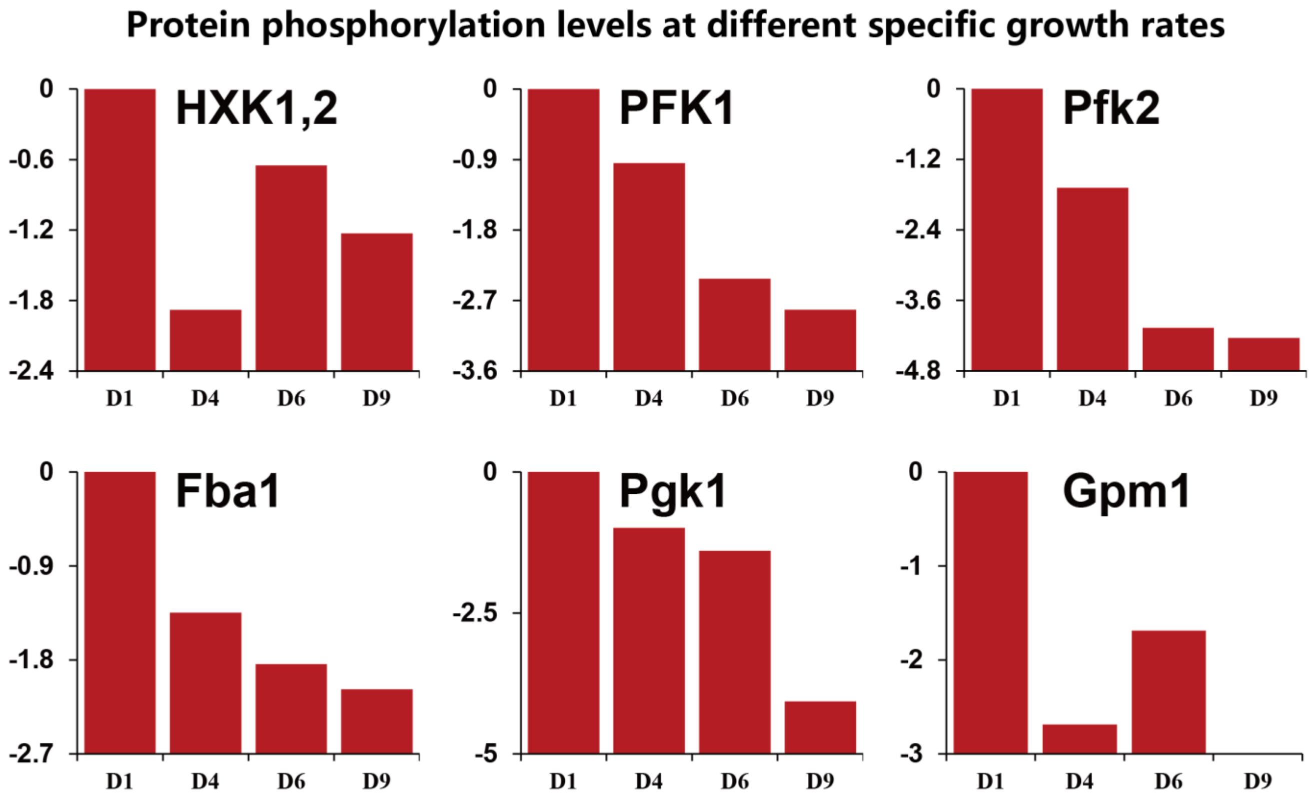
Protein phosphorylation levels at different specific growth rates
HXK1,2
PFK1
Pfk2
Fba1
Pgk1
Gpm1
0
-0.6
-1.2
-1.8
-2.4
-0.9
-2.7
-3.6
-4.8
-2.5
-5
-1
-2
-3
D1
D4
D6
D9

(C)

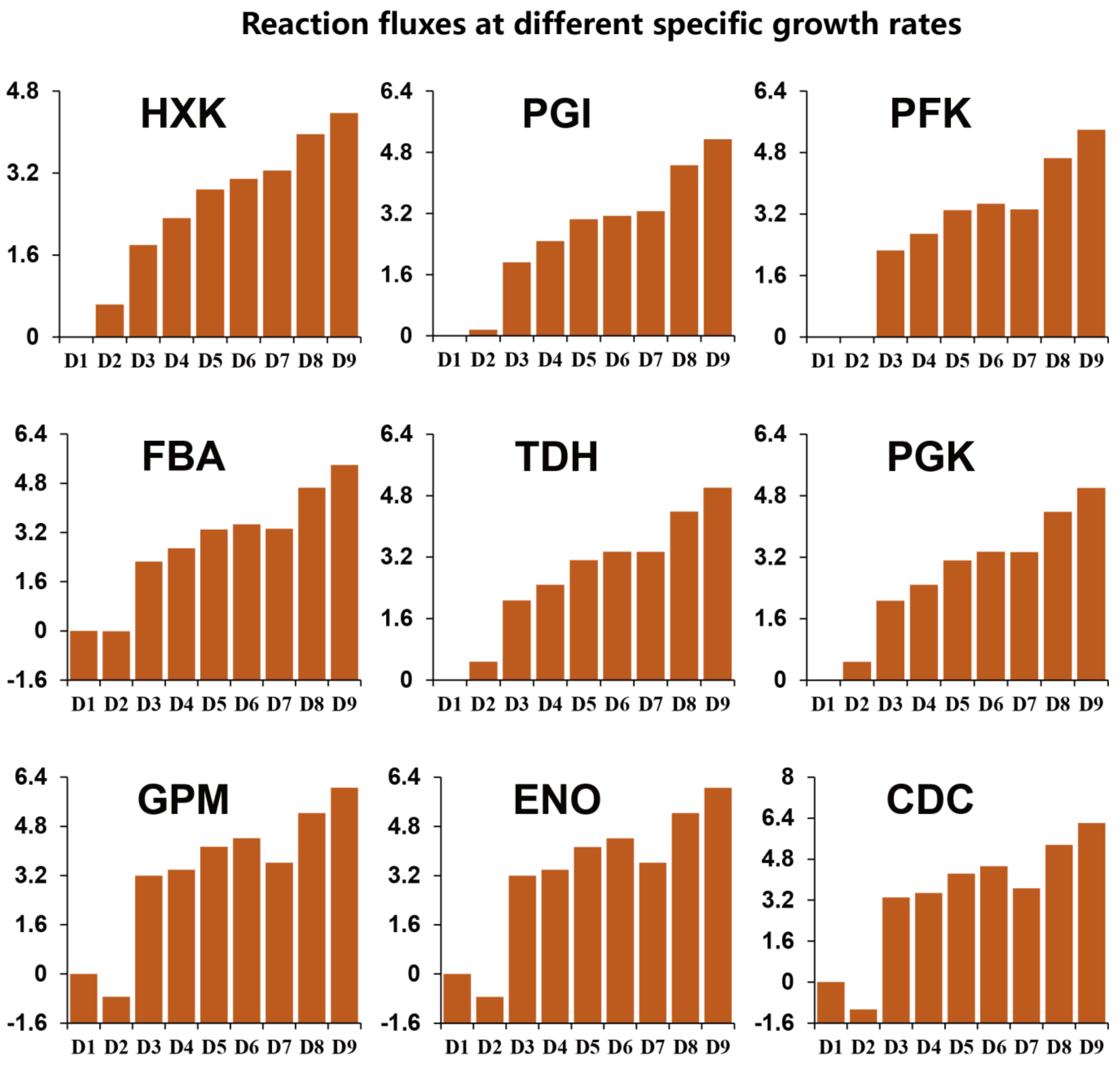

(D)

Supplementary Figure 9. Relative abundance of glycolysis pathway mRNA level, protein level, protein phosphorylation level, and reaction flux level. A) Detail plots of mRNA levels under 9 dilution rates of the glycolysis pathway; B) Detail plots of protein levels under 9 dilution rates of the glycolysis pathway; C) Detail plots of protein phosphorylation levels under 9 dilution rates of the glycolysis pathway; D) Detail plots of reaction fluxes under 9 dilution rates of the glycolysis pathway. Source data are provided as a Source Data file.

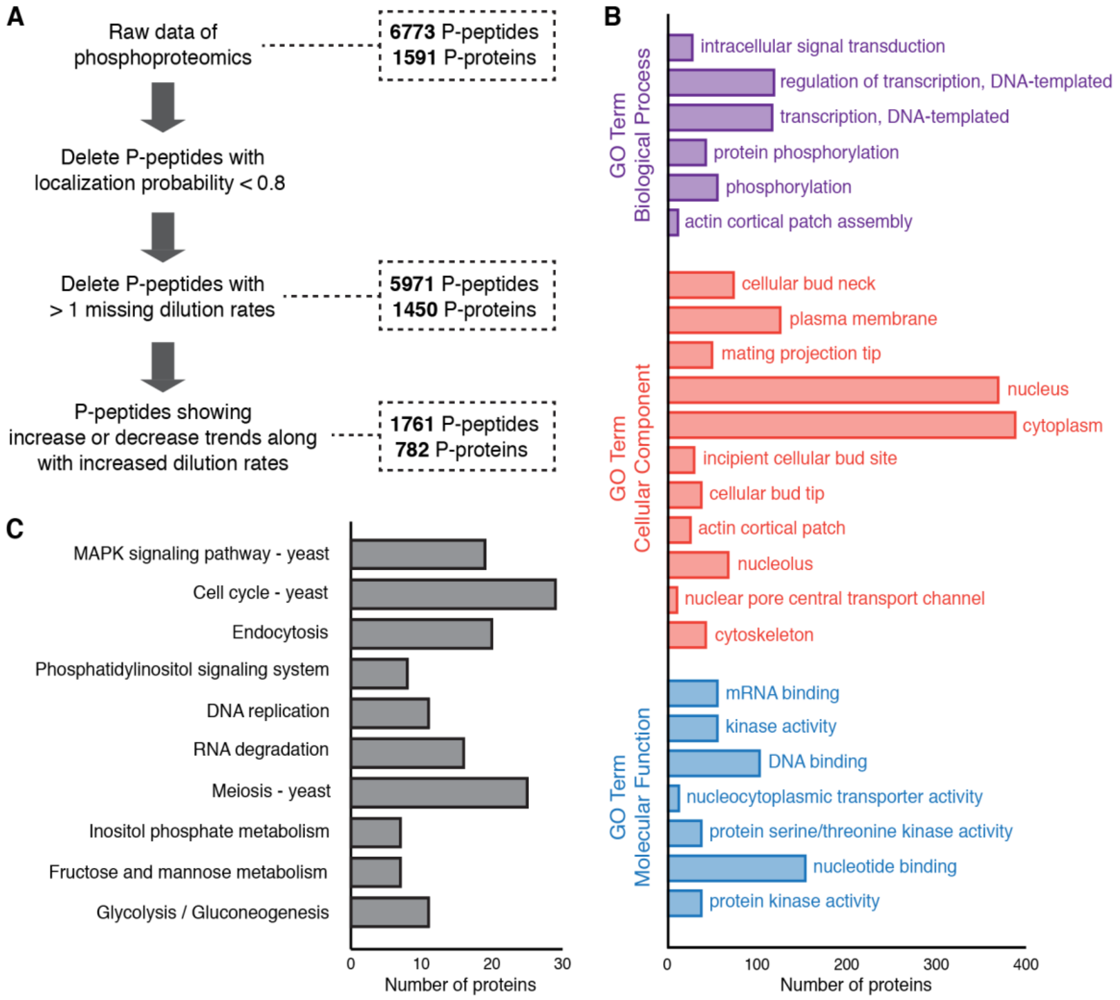

Supplementary Figure 10. Phosphoproteomics data analyzing method and enrichment analysis. (A) Workflow of phosphoproteomics data processing and number of phosphopeptides (P-peptides) and phosphoproteins (P-proteins) at each step. (B) Enrichment of P-proteins in Gene Ontology (GO) terms. Only the significantly (false discovery rate, FDR < 0.01) enriched GO terms are shown. (C) Enrichment of P-proteins in KEGG pathways. All the pathways are shown without being filtered by statistics. Source data are provided as a Source Data file.

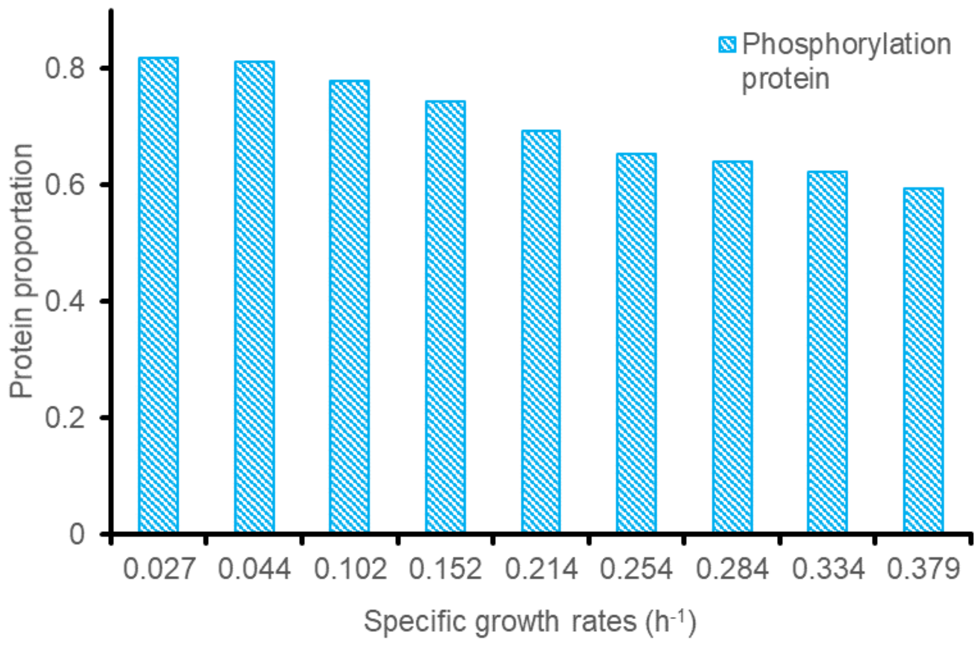

Supplementary Figure 11. Phosphorylation level of chaperone proteins studied across nine specific growth rates. Protein phosphorylation level were calculated by the ratio of phosphorylated protein to total protein of chaperon. It is clear that although proteins of chaperon are highly phosphorylated, a downward trend is shown with the increase of specific growth rate. Source data are provided as a Source Data file.

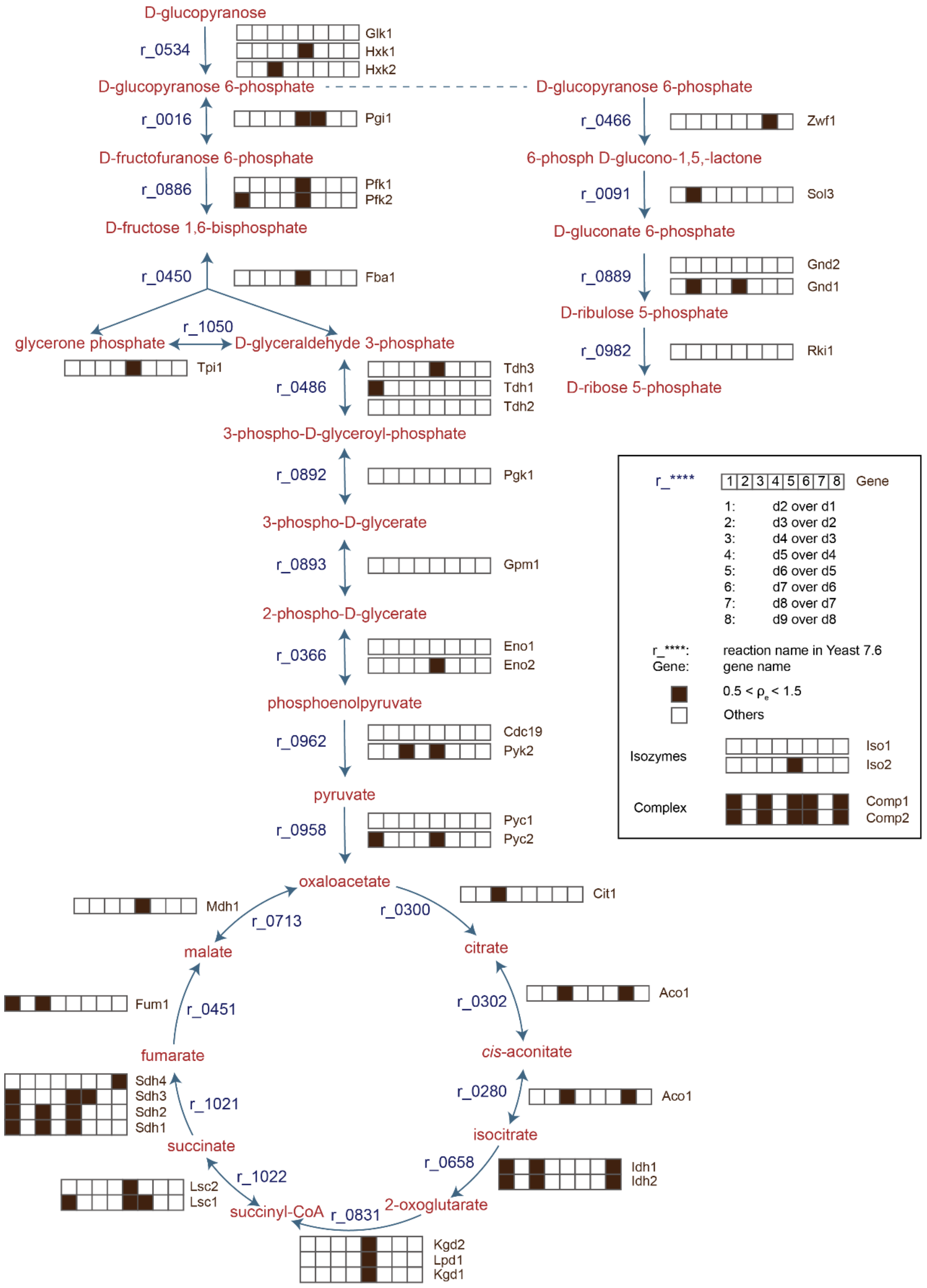

D-glucopyranose
r_0534
Glk1
Hxk1
Hxk2
D-glucopyranose 6-phosphate
r_0016
Pgi1
D-fructofuranose 6-phosphate
r_0886
Pfk1
Pfk2
D-fructose 1,6-bisphosphate
r_0450
Fba1
r_1050
glycerone phosphate
D-glyceraldehyde 3-phosphate
Tpi1
r_0486
Tdh3
Tdh1
Tdh2
3-phospho-D-glyceroyl-phosphate
r_0892
Pgk1
3-phospho-D-glycerate
r_0893
Gpm1
2-phospho-D-glycerate
r_0366
Eno1
Eno2
phosphoenolpyruvate
r_0962
Cdc19
Pyk2
pyruvate
r_0958
Pyc1
Pyc2
oxaloacetate
D-glucopyranose 6-phosphate
r_0466
Zwf1
6-phosph D-glucono-1,5,-lactone
r_0091
Sol3
D-gluconate 6-phosphate
r_0889
Gnd2
Gnd1
D-ribulose 5-phosphate
r_0982
Rki1
D-ribose 5-phosphate
r_****
1 2 3 4 5 6 7 8
Gene
1: d2 over d1
2: d3 over d2
3: d4 over d3
4: d5 over d4
5: d6 over d5
6: d7 over d6
7: d8 over d7
8: d9 over d8
r_****: reaction name in Yeast 7.6
Gene: gene name
0.5 < ρe < 1.5
Others
Isozymes
Iso1
Iso2
Complex
Comp1
Comp2
Mdh1
r_0713
malate
r_0300
Cit1
citrate
Fum1
r_0451
r_0302
Aco1
fumarate
cis-aconitate
Sdh4
Sdh3
Sdh2
Sdh1
r_1021
r_0280
Aco1
isocitrate
succinate
r_0658
Idh1
Idh2
Lsc2
Lsc1
r_1022
succinyl-CoA
r_0831
2-oxoglutarate
Kgd2
Lpd1
Kgd1

(A)

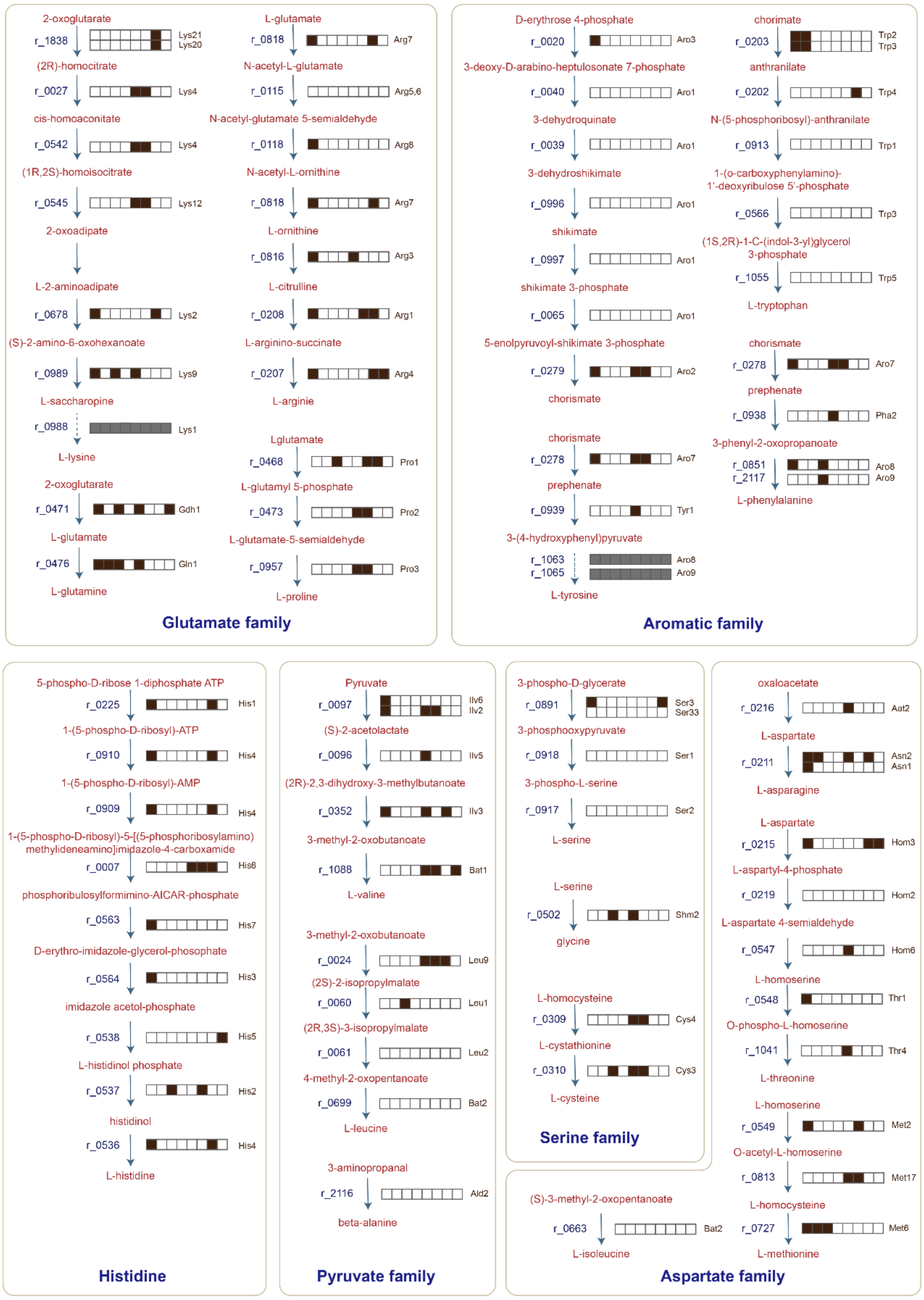

(B)

Supplementary Figure 12. Enzyme activity regulation events of central carbon metabolism and amino acid synthesis pathway. Hierarchical regulation analysis method was used to investigate enzymatic activity regulation events. The black block indicates that enzyme activity regulation events may exist, while the white block indicates that enzyme activity regulation events are almost non-existent.(A) Central carbon metabolic pathways; (B) Amino acid synthesis pathways.

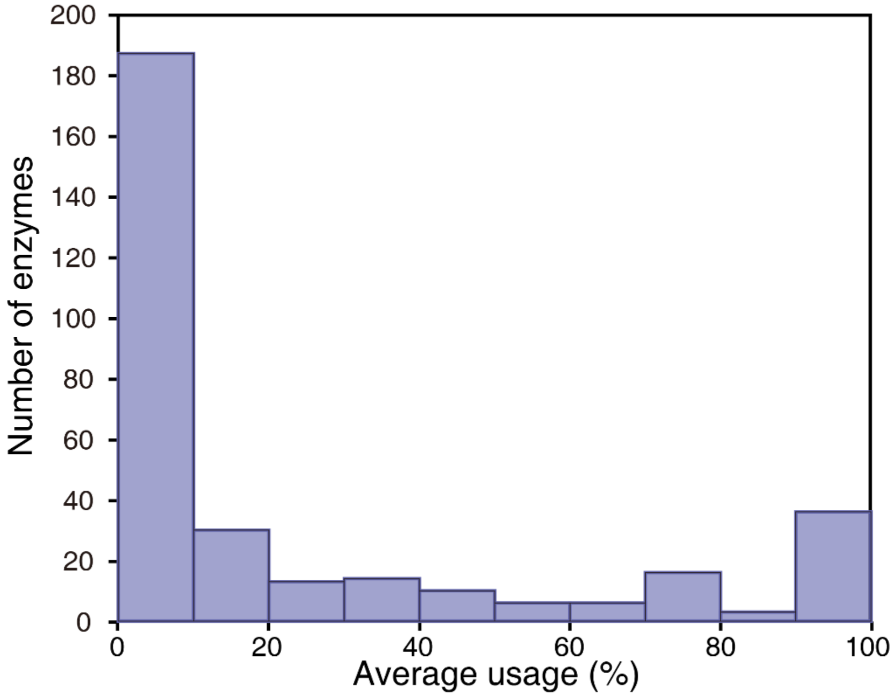

Supplementary Figure 13. Number of enzymes with different average enzyme usages (n=321) for enzyme usage analysis. Source data are provided as a Source Data file.

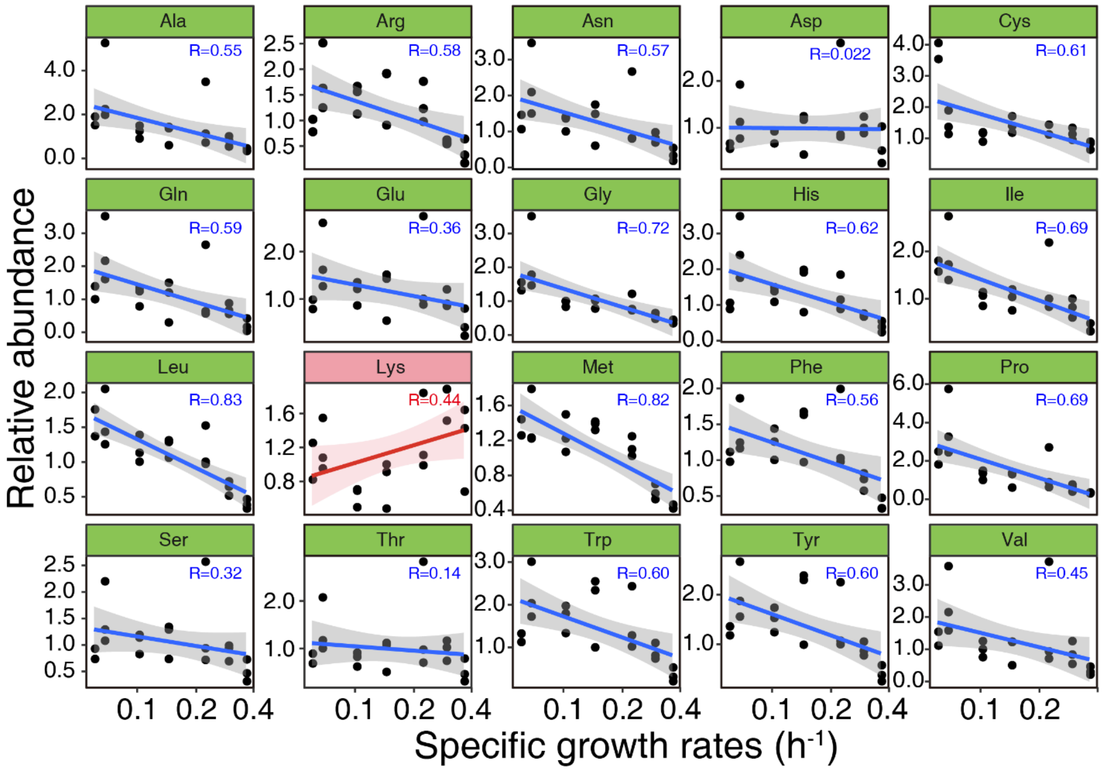

Supplementary Figure 14. Linear correlation regression of individual amino acids to specific growth rate. Among them, only lysine shows an increasing trend (marked in red). Shaded bands represents the 95% confidence intervals. Source data are provided as a Source Data file.

Supplementary Table 5. Correlation between proteins and reaction fluxes for aminoacyl-tRNA biosynthesis reactions, and multiple comparisons were corrected using FDR < 0.05.

Reaction	Name	Correlation coefficient	p-value	Corrected p-value
Alanyl-tRNA synthesis	YOR335C	0.767	0.0159	0.0152
Arginyl-tRNA synthesis	YDR341C	-0.650	0.0581	0.0339
Asparaginyl-tRNA synthesis	YHR019C	0.583	0.0992	0.0521
Aspartyl-tRNA synthesis	YLL018C	-0.683	0.0424	0.0327
Cysteinyl-tRNA synthesis	YNL247W	0.933	0.0002	0.0012
Glutaminyl-tRNA synthesis	YOR168W	0.917	0.0005	0.0013
Glutamyl-tRNA synthesis	YGL245W	0.850	0.0037	0.0043
Glycyl-tRNA synthesis	YBR121C	0.767	0.0159	0.0152
Isoleucyl-tRNA synthesis	YBL076C	0.600	0.0876	0.0484
Leucyl-tRNA synthesis	YPL160W	0.900	0.0009	0.0017
Lysyl-tRNA synthesis	YDR037W	0.650	0.0580	0.0339
Methionyl-tRNA synthesis	YGR264C	0.917	0.0005	0.0013
Phenylalanyl-tRNA synthesis	YFL022C[a]	0.867	0.0025	0.0037
Phenylalanyl-tRNA synthesis	YLR060W[a]	0.933	0.0002	0.0012
Prolyl-tRNA synthesis	YHR020W	0.900	0.0009	0.0017
Seryl-tRNA synthesis	YDR023W[b]	0.667	0.0499	0.0327
Seryl-tRNA synthesis	YHR011W[b]	0	1	0.5
Threonyl-tRNA synthesis	YIL078W	0.667	0.0499	0.0327
Tryptophanyl-tRNA synthesis	YOL097C	0.850	0.0037	0.0043
Tyrosyl-tRNA synthesis	YGR185C	0.667	0.0499	0.0327
Valyl-tRNA synthesis	YGR094W	0.717	0.0298	0.0261

Note: a. YFL022C and YLR060W code the two components phenylalanyl-tRNA synthetase complex; b. YDR023W and YHR011W code isozymes of Seryl-tRNA synthetase, respectively.

Supplementary Table 6. Total protein contents measured under all nine specific growth rates. Triplicates were used to calculate the standard deviations for each sample.

Specific growth rate (h^{-1}, AVG±SD)	0.027 ±0.002	0.044 ±0.001	0.102 ±0.001	0.152 ±0.003	0.214 ±0.011	0.254 ±0.003	0.284 ±0.013	0.334 ±0.009	0.379 ±0.011
**Total protein content (%, AVG±SD)**	29.9 ±0.3	31.2 ±0.2	37.1 ±0.7	41.9 ±2.4	32.0 ±4.8	37.8 ±0.8	39.1 ±0.9	41.9 ±0.7	39.1 ±6.5

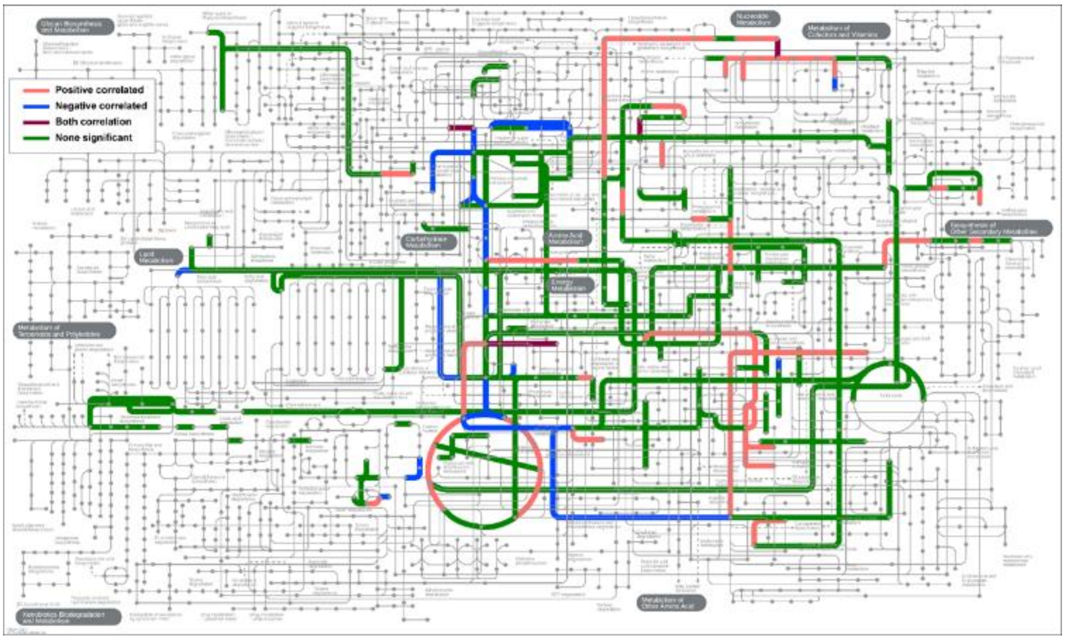

Supplementary Figure 15. Reaction map showing correlations between both mRNA/flux and protein/flux pairs. Information is plotted using iPath3 tool. In the map, reactions with positive correlations are shown as pink lines, and that reactions with negative correlations as blue lines, reactions with both positive and negative correlations as red lines, other non-significantly correlated reactions shown as green lines.

Supplementary References

1. Warner JR. The assembly of ribosomes in yeast. *The Journal of Biological Chemistry* **246**, 447-454 (1971).

2. Milo R, Jorgensen P, Moran U, Weber G, Springer M. BioNumbers--the database of key numbers in molecular and cell biology. *Nucleic Acids Res* **38**, D750-D753, BNID 100986 (2010).

3. Waldron C, Lacroute F. Effect of growth rate on the amounts of ribosomal and transfer ribonucleic acids in yeast. *J Bacteriol* **122**, 855-865 (1975).

4. Saurez-Mendez CA, Hanemaaijer M, Pierick At, Wolters JC, Heijnen JJ, Wahl SA. Interaction of storage carbohydrates and other cyclic fluxes with central metabolism: A quantitative approach by non-stationary ^{13}C metabolic flux analysis. *Metabolic Engineering Communications* **3**, 52-63 (2016).

5. B.Canelas A, Ras C, Pierick At, Gulik WMv, Heijnen JJ. An *in vivo* data-driven framework for classification and quantification of enzyme kinetics and determination of apparent thermodynamic data. *Metabolic Engineering* **13**, 294-306 (2011).

6. Hackett SR*, et al.* Systems-level analysis of mechanisms regulating yeast metabolic flux. *Sci* **354**, aaf2786 (2016).

7. Holland P, Bergenholm D, Borlin CS, Liu G, Nielsen J. Predictive models of eukaryotic transcriptional regulation reveals changes in transcription factor roles and promoter usage between metabolic conditions. *Nucleic Acids Res* **47**, 4986-5000 (2019).

8. Sierkstra LN, Verbakel JM, Verrips CT. Analysis of transcription and translation of glycolytic enzymes in glucose-limited continuous cultures of *Saccharomyces cerevisiae*. *Journal of General Microbiology* **138**, 2559-2566 (1992).

9. Lee TI*, et al.* Transcriptional regulatory networks in *Saccharomyces cerevisiae*. *Sci* **298**, 799-804 (2002).

10. Lahtvee P-J*, et al.* Absolute Quantification of Protein and mRNA Abundances Demonstrate Variability in Gene-Specific Translation Efficiency in Yeast. *Cell Systems* **4**, 495-504.e495 (2017).
