# Supplementary material for: Proteome allocations change linearly with the specific growth rate of Saccharomyces cerevisiae under glucose limitation
Source: Nat Commun. 2022 May 20;13:2819. doi: 10.1038/s41467-022-30513-2 (PMC9122918; doi:10.1038/s41467-022-30513-2)
Supplement: Supplementary file 3 — Description of Additional Supplementary Files [file 41467_2022_30513_MOESM3_ESM.docx]

**File Name: Supplementary Software**

**Description:** Python or R codes for the statistical analysis of proteome and transcriptome data, correlation analysis of protein/mRNA vs specific growth rate, ribosome protein related information statistics, Bayes inference for detecting allosteric regulation inferring. More details are provided in the corresponding readme files.

**File Name:** **Supplementary Data 1**

**Description:** Main absolute proteome data generated in this study. There are 27 samples in the whole study and corresponding to S1-S27 column. We carried out nine dilution rates chemostat experiments and triplicate samples were gathered for each dilution rate, so you can see nine specific growth rate ranging from μ= 0.027 h^-1^ to μ = 0.379 h^-1^. Protein names are given in column “Majority protein IDs” corresponding coding gene is given in column “Gene Name”, with their belonging functional categories given in column “Categories”. Protein abundance values are given with unit of molecules per cell.

**File Name:** **Supplementary Data 2**

**Description:** Categories of proteins classified in this work based on Metzl-Raz E. *et al.*, 2017, eLife, 6, e28034 (doi: 10.7554/eLife.28034). For each category one specific sheet with the same name of the category is given. Proteins that could not be categorized into the 11 categories are listed as a single sheet named “Others”.

**File Name:** **Supplementary Data 3**

**Description:** Main absolute transcriptome data generated in this study. mRNA names are given in column “mRNA”, absolute mRNA concentrations for each dilution rate are given in each columns headed with the specific growth rate of the corresponding chemostats that ranges from μ= 0.027 h^-1^ to μ = 0.379 h^-1^. mRNA absolute concentrations are given with unit of molecules per cell.

**File Name:** **Supplementary Data 4**

**Description:** Data for calculating the average amino acid molecular weight based on amino acid compositions under each of the nine chemostats studied in this work. Amount of each amino acid under all the nine chemostats was calculated based on each protein’s amino acid composition and absolute concentration of the proteins, then the average amino acid molecular weight was estimated by averaging all amino acids average molecular weight under all chemostat conditions.
