# Supplementary material for: Proteome allocations change linearly with the specific growth rate of Saccharomyces cerevisiae under glucose limitation
Source: Nat Commun. 2022 May 20;13:2819. doi: 10.1038/s41467-022-30513-2 (PMC9122918; doi:10.1038/s41467-022-30513-2)
Supplement: Supplementary file 8 — Supplementary Software [file 41467_2022_30513_MOESM8_ESM.zip › NCOMMS-21-15807B_supp-soft/Code_01-Proteome data processing/ReadMe.docx]

| **File** | **Short description** |
| --- | --- |
| processRawToGenerateProteomeData.R | R code designed to generate the absolute proteome concentration based on raw data files, which are described as follows. |
| IS-S288C.csv | Output file of MaxQuant containing MS intensity of ^15^N,^13^C-lysine labeled protein internal standard samples. |
| ProteinGroupsS288C.csv | Output file of MaxQuant containing MS intensity of normal samples. |
| TotalProteinMeasured.csv | Total protein content measured under all nine chemostats conditions. |

**Further explanation:** processRawToGenerateProteomeData.R is written using R, and detail description of the code is coded as comments in the source file.
