# Supplementary material for: Proteome allocations change linearly with the specific growth rate of Saccharomyces cerevisiae under glucose limitation
Source: Nat Commun. 2022 May 20;13:2819. doi: 10.1038/s41467-022-30513-2 (PMC9122918; doi:10.1038/s41467-022-30513-2)
Supplement: Supplementary file 8 — Supplementary Software [file 41467_2022_30513_MOESM8_ESM.zip › NCOMMS-21-15807B_supp-soft/Code_02_Correlation_analysis_between_prteins_and_mRNAs/ReadMe.docx]

| **File** | **Short description** |
| --- | --- |
| Correlation_between_protein_and_mRNA.ipynb | This script is written with jupyter notebook, and depends on pvsm_new.xlsx, which is explained as follows. |
| pvsm_new.xlsx | Input file for the above script, which contains both absolute proteome and transcriptome data. |
| corrected_pvsm_correlation_new20210925.csv | Generated results by the described script. |
| package-list.txt | Anaconda environment packages and versions that used to develop the code. All following python codes used the same environment. |

**Further explanation:** Correlation_between_protein_and_mRNA.ipynb is written with jupyter notebook, choose a location where you put the input file pvsm_new.xlsx, and open the script using jupyter notebook, the running environment for the author is listed in package-list.txt.
