# Supplementary material for: Proteome allocations change linearly with the specific growth rate of Saccharomyces cerevisiae under glucose limitation
Source: Nat Commun. 2022 May 20;13:2819. doi: 10.1038/s41467-022-30513-2 (PMC9122918; doi:10.1038/s41467-022-30513-2)
Supplement: Supplementary file 8 — Supplementary Software [file 41467_2022_30513_MOESM8_ESM.zip › NCOMMS-21-15807B_supp-soft/Code_03_Prteome_and_Transcriptome_normality_check/ReadMe.docx]

| **File** | **Short description** |
| --- | --- |
| protein_mRNA_normality_check.ipynb | This script is designed to check the normality distribution of both proteome and transcriptome data, written with jupyter notebook, and depends on pvsm_new.xlsx, which is explained as follows. |
| proteome_data_20201007 | Input file for the above script, which contains absolute proteome data. |
| Transcripteome20210906.csv | Input file for the above script, which contains absolute transcriptome data. |

**Further explanation:** protein_mRNA_normality_check.ipynb is written with jupyter notebook, choose a location where you put the input files, and open the script using jupyter notebook. The running environment for the author is listed in in description of Code_02.
