# Supplementary material for: Proteome allocations change linearly with the specific growth rate of Saccharomyces cerevisiae under glucose limitation
Source: Nat Commun. 2022 May 20;13:2819. doi: 10.1038/s41467-022-30513-2 (PMC9122918; doi:10.1038/s41467-022-30513-2)
Supplement: Supplementary file 8 — Supplementary Software [file 41467_2022_30513_MOESM8_ESM.zip › NCOMMS-21-15807B_supp-soft/Code_04_Leave-one-out-testing/ReadMe.docx]

| **File** | **Short description** |
| --- | --- |
| Leave_one_out_test.py | This script is designed to do leave-one-out test for each category determining whether a specific protein determines the correlation results of the group and specific growth rate. |
| ProteinAbsConcentration20210924.xlsx | Input file for the above script, which contains absolute proteome data. |
| eLIFE-ProteinCategoriesModify20200527.xlsx | Input file for the above script, which contains all 11 protein functional group definitions. |

**Further explanation:** Leave_one_out_test.py is written with jupyter notebook, choose a location where you put the input files, and open the script using jupyter notebook. The running environment for the author is listed in in description of Code_02.
