# Supplementary material for: Proteome allocations change linearly with the specific growth rate of Saccharomyces cerevisiae under glucose limitation
Source: Nat Commun. 2022 May 20;13:2819. doi: 10.1038/s41467-022-30513-2 (PMC9122918; doi:10.1038/s41467-022-30513-2)
Supplement: Supplementary file 8 — Supplementary Software [file 41467_2022_30513_MOESM8_ESM.zip › NCOMMS-21-15807B_supp-soft/Code_05_Correlation_between_individual_protein_and_specific_growth_rate/ReadMe.docx]

| **File** | **Short description** |
| --- | --- |
| Correlation for individual protein vs growth rate.ipynb | This script will do the correlation analysis between protein and cell specific grwoth rate for individual gene, and depends on pvsm_new.xlsx, which is explained as follows. |
| pvsm_new.xlsx | Input file for the above script, which contains absolute proteome data. |

**Further explanation:** Correlation for individual protein vs growth rate.ipynb is written with jupyter notebook, choose a location where you put the input files, and open the script using jupyter notebook. The running environment for the author is listed in in description of Code_02.
