# Supplementary material for: Proteome allocations change linearly with the specific growth rate of Saccharomyces cerevisiae under glucose limitation
Source: Nat Commun. 2022 May 20;13:2819. doi: 10.1038/s41467-022-30513-2 (PMC9122918; doi:10.1038/s41467-022-30513-2)
Supplement: Supplementary file 8 — Supplementary Software [file 41467_2022_30513_MOESM8_ESM.zip › NCOMMS-21-15807B_supp-soft/Code_06_CorrelationBetweenFluxAndProteinAndmRNA/ReadMe.docx]

| **File** | **Short description** |
| --- | --- |
| doCorrelationAnalysisFlux_mRNA_Protein.py | This script is designed for dealing with the correlation analysis between reaction flux and mRNA level or protein level, only catabolic enzyme coding genes that carry reaction flux get involved in the analysis, and depends on the following excel file. |
| FluxTranscriptomeAndProteomeCorrelationData.xlsx | Input file for the above script, which contains yeast 7.6 GSMM reaction model, and corresponding reaction fluxes and proteome and transcriptome data. |

**Further explanation:** doCorrelationAnalysisFlux_mRNA_Protein.py is written with python 3.6, choose a location where you put the input files, and run the script. The running environment for the author is listed in in description of Code_02.
