# Supplementary material for: Proteome allocations change linearly with the specific growth rate of Saccharomyces cerevisiae under glucose limitation
Source: Nat Commun. 2022 May 20;13:2819. doi: 10.1038/s41467-022-30513-2 (PMC9122918; doi:10.1038/s41467-022-30513-2)
Supplement: Supplementary file 8 — Supplementary Software [file 41467_2022_30513_MOESM8_ESM.zip › NCOMMS-21-15807B_supp-soft/Code_07_Lysine_and_Arginine_pathway_enzymes_correlation_with_ribosome_proteins/ReadMe.docx]

| **File** | **Short description** |
| --- | --- |
| Lysine_Arginine_pathway_analysis.py | This script is designed to analysis the correlation of Lysine/Arginine biosynthesis  pathway proteins to that of Ribosome proeins, and depends on pvsm_new.xlsx and eLIFE-ProteinCategoriesModify20200527.xlsx. |
| eLIFE-ProteinCategoriesModify20200527.xlsx | Input file for the above script, which contains 11 protein functional groups defination. |
| pvsm_new.xlsx | Input file for the above script, which contains absolute proteome and transcriptome data. |

**Further explanation:** protein_mRNA_normality_check.ipynb is written with python 3.6, choose a location where you put the input files, and run the script. The running environment for the author is listed in in description of Code_02.
