# Supplementary material for: Proteome allocations change linearly with the specific growth rate of Saccharomyces cerevisiae under glucose limitation
Source: Nat Commun. 2022 May 20;13:2819. doi: 10.1038/s41467-022-30513-2 (PMC9122918; doi:10.1038/s41467-022-30513-2)
Supplement: Supplementary file 8 — Supplementary Software [file 41467_2022_30513_MOESM8_ESM.zip › NCOMMS-21-15807B_supp-soft/Code_08_Ribosome_amino_acid_statistics/Readme.docx]

| **File** | **Short description** |
| --- | --- |
| ribosomeProtSeqStatistics.py | This script use the biopython package to read and do statistic study on the yeast ribosome protein sequences, and depends on the following two files |
| sgdSystemID2SWISSPROT.xlsx | Input file for the above script, which contains name conversion information for sgd system ID to SWISS PROT id. |
| sgd.fasta.gz | Input file for the above script, which contains all protein sequence for Saccharomyces cerevisiae. |

**Further explanation:** ribosomeProtSeqStatistics.py is written with python 3.6, choose a location where you put the input files, and run the script. The running environment for the author is listed in in description of Code_02.
