# Supplementary material for: Proteome allocations change linearly with the specific growth rate of Saccharomyces cerevisiae under glucose limitation
Source: Nat Commun. 2022 May 20;13:2819. doi: 10.1038/s41467-022-30513-2 (PMC9122918; doi:10.1038/s41467-022-30513-2)
Supplement: Supplementary file 8 — Supplementary Software [file 41467_2022_30513_MOESM8_ESM.zip › NCOMMS-21-15807B_supp-soft/Code_09_Correlation_between_protein_and_aminotacyl-tRNA_biosynthesis/ReadMe.docx]

| **File** | **Short description** |
| --- | --- |
| Correlation_between_protein_and_tRNAsynthesis.py | This script is designed to do correlation analysis between enzymes concentration and Aminoacyl-tRNA synthesis reactions flux, and depends on pvsm_new.xlsx and tRNA_Fluxes_proteome.xlsx |
| tRNA_Fluxes_proteome.xlsx | Input file for the above script, which contains all aminoacyl-tRNA synthesis reaction fluxes under all nine chemostat conditions. |

**Further explanation:** Correlation_between_protein_and_tRNAsynthesis.py is written with python 3.6, choose a location where you put the input files, and run the script. The running environment for the author is listed in in description of Code_02.
