# Supplementary material for: Proteome allocations change linearly with the specific growth rate of Saccharomyces cerevisiae under glucose limitation
Source: Nat Commun. 2022 May 20;13:2819. doi: 10.1038/s41467-022-30513-2 (PMC9122918; doi:10.1038/s41467-022-30513-2)
Supplement: Supplementary file 8 — Supplementary Software [file 41467_2022_30513_MOESM8_ESM.zip › NCOMMS-21-15807B_supp-soft/Code_10_Pearson correlation analysis functional groups level/ReadMe.docx]

| **File** | **Short description** |
| --- | --- |
| Correlation_analysis_between_proteome_and_transcriptome_group_fractions.py | This script is used to check correlation between proteome and transcriptome in functional groups, and depends on the input file list bellow |
| Group_comparison_between_proeome_and_transcriptome.xlsx | Input file for the above script, which contains both proteome and transcriptome fractions for individual functional groups |

**Further explanation:**

Correlation_analysis_between_proteome_and_transcriptome_group_fractions.py is written with python 3.6, choose a location where you put the input file, and run the script. The running environment for the author is listed in in description of Code_02.
