# Supplementary material for: Proteome allocations change linearly with the specific growth rate of Saccharomyces cerevisiae under glucose limitation
Source: Nat Commun. 2022 May 20;13:2819. doi: 10.1038/s41467-022-30513-2 (PMC9122918; doi:10.1038/s41467-022-30513-2)
Supplement: Supplementary file 8 — Supplementary Software [file 41467_2022_30513_MOESM8_ESM.zip › NCOMMS-21-15807B_supp-soft/Code_11-BayesianInferenceScript/ReadMe.docx]

| **File** | **Short description** |
| --- | --- |
| LogLinearKineticsModelBayesInference.py | This script is designed to do Bayesian inference of kinetic models for identifying allosteric regulators with metabolome and fluxome data, written with python 3.6, and depends on CChemostatsBayesModelData-withmacro.xlsm. |
| CChemostatsBayesModelData-withmacro.xlsm | Input file for the above script, which contains central carbon metabolism reaction models and corresponding reaction fluxes and metabolome data. |

**Further explanation:** LogLinearKineticsModelBayesInference.py is written with python 3.6, choose a location where you put the input file, and run the script. The running environment for the author is listed in in description of Code_02. Detailed explanation of the code can be found in the code’s comments part.
